# Respiratory muscle ultrasonography evaluation and its clinical application in stroke patients: A review

**DOI:** 10.3389/fnins.2023.1132335

**Published:** 2023-04-06

**Authors:** Xiaoman Liu, Ying Yang, Jie Jia

**Affiliations:** ^1^Department of Rehabilitation Medicine, The People’s Hospital of Suzhou New District, Suzhou, China; ^2^Department of Rehabilitation Medicine, Fudan University Huashan Hospital, Shanghai, China; ^3^National Center for Neurological Disorders, Shanghai, China; ^4^National Clinical Research Center for Aging and Medicine, Fudan University Huashan Hospital, Shanghai, China

**Keywords:** diaphragm, evaluation, ultrasonography, stroke, respiratory muscle

## Abstract

**Background:**

Respiratory muscle ultrasound is a widely available, highly feasible technique that can be used to study the contribution of the individual respiratory muscles related to respiratory dysfunction. Stroke disrupts multiple functions, and the respiratory function is often significantly decreased in stroke patients.

**Method:**

A search of the MEDLINE, Web of Science, and PubMed databases was conducted. We identified studies measuring respiratory muscles in healthy and patients by ultrasonography. Two reviewers independently extracted and documented data regarding to the criteria. Data were extracted including participant demographics, ultrasonography evaluation protocol, subject population, reference values, etc.

**Result:**

A total of 1954 participants from 39 studies were included. Among them, there were 1,135 participants from 19 studies on diaphragm, 259 participants from 6 studies on extra-diaphragmatic inspiratory muscles, and 560 participants from 14 studies on abdominal expiratory muscles. The ultrasonic evaluation of diaphragm and abdominal expiratory muscle thickness had a relatively typically approach, while, extra-diaphragmatic inspiratory muscles were mainly used in ICU that lack of a consistent paradigm.

**Conclusion:**

Diaphragm and expiratory muscle ultrasound has been widely used in the assessment of respiratory muscle function. On the contrary, there is not enough evidence to assess extra-diaphragmatic inspiratory muscles by ultrasound. In addition, the thickness of the diaphragm on the hemiplegic side was lower than that on the non-hemiplegic side in stroke patients. For internal oblique muscle (IO), rectus abdominis muscle (RA), transversus abdominis muscle (TrA), and external oblique muscle (EO), most studies showed that the thickness on the hemiplegic side was lower than that on the non-hemiplegic side.

**Clinical Trial Registration**: The protocol of this review was registered in the PROSPERO database (CRD42022352901).

## 1. Introduction

The respiratory muscle pump consists of three primary groups controlling ventilation: the primary muscle of inspiration, the accessory inspiratory, and the expiratory muscles (Shi et al., 2021). The primary muscle of inspiration is the diaphragm, a thin dome-shaped muscle positioned between the chest and abdomen. As the most important respiratory muscle, the diaphragm contributes 60–80% of the ventilation needs of the human body. However, the diaphragm is not the only inspiratory muscle involved in ventilation. When the load imposed on the diaphragm increases, the accessory inspiratory muscles, such as the parasternal intercostal muscles, external intercostal muscles, scalene muscles, and sternocleidomastoid muscles, are recruited to assist in inspiration (Tuinman et al., 2020). With further loading, the expiratory muscles are activated in a fixed hierarchy to assist expiration (Shi et al., 2019). The role of expiratory muscle includes reducing end-expiratory lung volume, reducing transpulmonary pressure, and increasing inspiratory muscle volume (Shi et al., 2019). There are also studies (Dres et al., 2020) showing that the muscle fibers of the parasternal intercostal muscles contract during inspiration, which expands the thoracic cavity, thereby increasing the tidal volume. Generally, during tidal ventilation, the diaphragm works in synergy with the scalene and external intercostal muscles to trigger inspiration, as well as with the dilator muscles of the upper airway. In cases of respiratory distress, the sternocleidomastoid muscles and the trapezius are also recruited (Vivier and Mekontso Dessap, 2020).

Ultrasonography can assess the mechanics, thickness, and strength of all the respiratory muscles (Matamis et al., 2013), and it may be possible to provide valuable information in this context to complement clinical examination. Recent studies proved that ultrasound permits the quantitative assessment of the excursion and thickness of the respiratory muscles to quantify their function (Cala et al., 1998; Sferrazza Papa et al., 2016; Yoshida et al., 2019; Bedewi et al., 2021; Kang et al., 2021). Different ultrasonic techniques have been validated through several studies (Adigozali et al., 2016; Tahan et al., 2016; Bedewi et al., 2021). Stroke disrupts multiple functions (Benditt and Boitano, 2013). The respiratory function is often significantly decreased in stroke patients, and the respiratory intensity is only about 50% of the normal population. The respiratory dysfunction can be attributed to the affected respiratory central nervous system and respiratory muscles (Rochester and Mohsenin, 2002; Jandt et al., 2011). In this context, the present review has two objectives. The first is to review the ultrasound assessment of respiratory muscles. The second objective is the clinical application of respiratory muscle ultrasound in stroke patients.

## 2. Methods

### 2.1. Selection of studies

**Figure 1:**
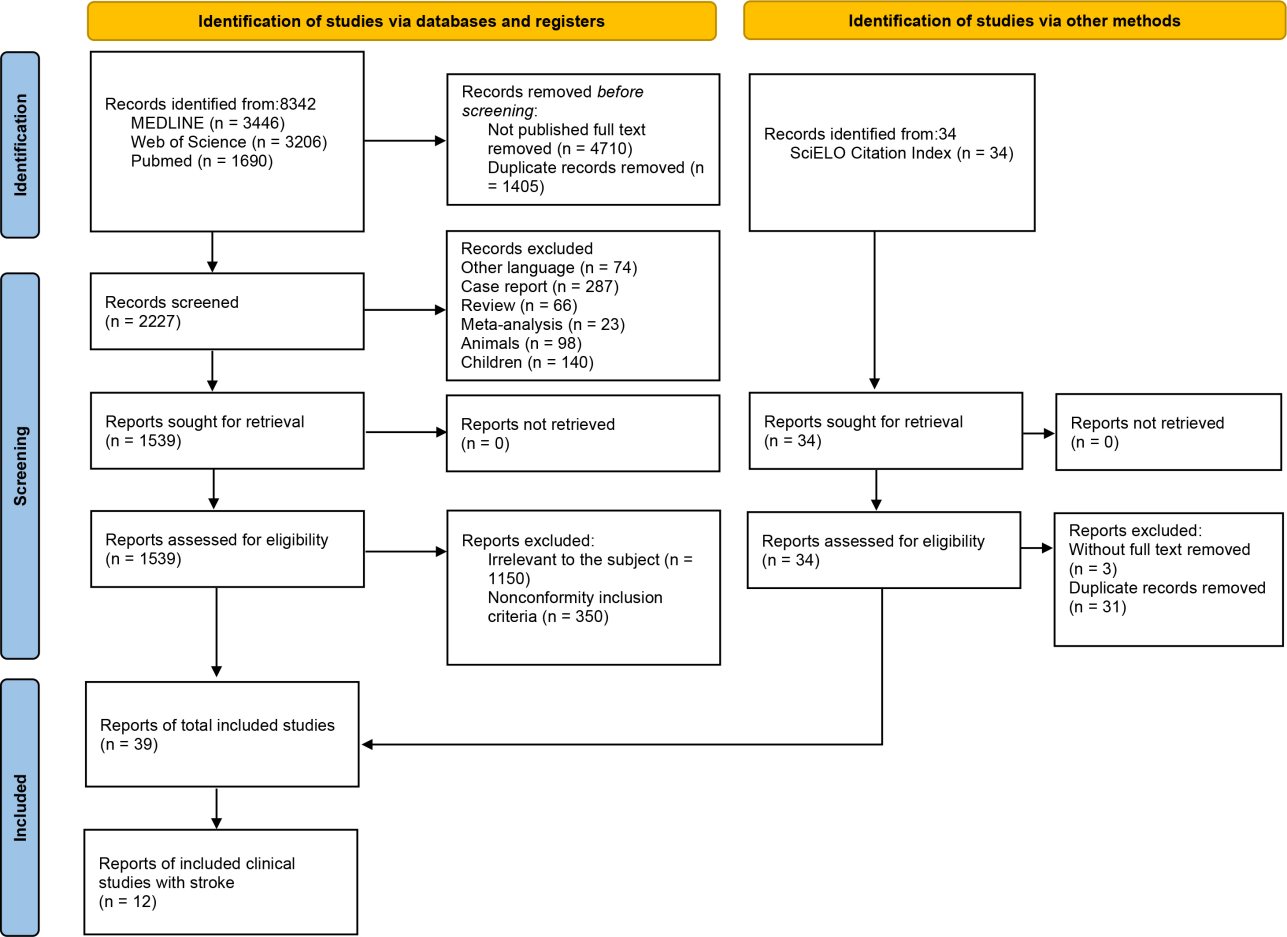
Flow diagram of the review.

The review was according to the PRISMA 2020 flow diagram (Figure 1 and Supplementary material 3). We intended to identify studies measuring respiratory muscles in healthy and patients by ultrasonography. Based on the suggestions of thoracic ultrasound from European Respiratory Society Statement (Laursen et al., 2021) and previous review on ultrasonography measurement (Sferrazza Papa et al., 2016), we chose studies based on the following criteria: (1) Study subjects were healthy or patients with respiratory muscle disorders for various causes, the subjects included in the part of the clinical application were stroke patients. (2) The ultrasonography evaluation of the respiratory muscle was clearly described with integrated protocol, the ultrasound equipment, experience of the operator, probe frequency selection, subject’s measurement position, selection of measuring points/ultrasound probe placement position, respiratory status during measurement, and whether the subject cooperated, and respiratory muscle’s values were recorded. (3) All included studies were published in English with full text (English articles that were not published in full text were removed), including original studies and clinical trials.

### 2.2. Data source and search strategy

We searched articles published in the MEDLINE, Web of Science, and PubMed databases with no date restrictions up to 19 November 2022 (Supplementary material 1). The following keywords were used: Respiratory muscle ultrasonography-related words: ultrasonography, ultrasound, echotomography, sonography, echography, diaphragm, respiratory muscles, ventilatory muscles, and intercostal muscles based on title, abstract, and MeSH terms, and trapezius, sternocleidomastoid muscles, scalene muscles, parasternal intercostal muscle, inspiratory muscle, and expiratory muscle based on title and abstract. Stroke-related words were stroke, hemiplegia, cerebrovascular, CVA, apoplexy, vascular, brain, cerebral, intracerebral, hemorrhage, infarct, and ischemia based on title, abstract, and MeSH terms. The search strategy was created based on the PICO strategy: P (patient)—adults (healthy or diseases); I (intervention)—ultrasonography of the respiratory muscles (e.g., diaphragm, extra-diaphragmatic inspiratory muscles, abdominal muscles); C (comparator)—none; O (outcomes)—methodology and reference value.

### 2.3. Literature review and data extraction

Two review authors independently read the titles and abstracts of all literatures searched by the databases. Unified standards were used to extract relevant information from the full text of articles that met the inclusions. Data were extracted including participant demographics, ultrasonography evaluation protocol, subject population, reference values, etc.

## 3. Ultrasonography evaluation of respiratory muscles

### 3.1. Inspiratory muscle

#### 3.1.1. Diaphragm

The diaphragm is a dome-shaped, fibromuscular partition between the thoracic and abdominal cavities (Gerscovich et al., 2001) that composed of four components: the transverse septum, pleuroperitoneal folds, esophageal mesentery, and muscular body wall laterally that separates the chest from the abdominal cavity (Sarwal et al., 2013). It is the most important inspiratory muscle and plays a major role in maintaining ventilation (Sferrazza Papa et al., 2016). Diaphragmatic ultrasound has been widely used in healthy individuals (Ueki et al., 1995) and clinical practice (Sferrazza Papa et al., 2016), and a wide range of normal and abnormal values has been reported (Gottesman and McCool, 1997; Boussuges et al., 2009; Sferrazza Papa et al., 2016). In general, there are two major forms of ultrasonographic assessment of the diaphragm: diaphragm excursion and thickness (Supinski et al., 2018).

Diaphragm excursion measurement (Table 1): 2–5 MHz transducer with M-mode ultrasonography is used to measure the diaphragm excursion. The participants are in the supine or standing position during ultrasonic measurement. Usually, the liver is used as a window on the right side, and the diaphragm is examined from the anterior subcostal approach (the right costal margin between the midclavicular and anterior axillary lines). While the spleen is used for the left side, the diaphragm was examined from a subcostal or low intercostal approach (between the anterior axillary and mid-axillary lines). One study has shown that supine position is preferred for the study of diaphragmatic excursion because of less variability in observations (Gerscovich et al., 2001). Visualization of the left diaphragm is more difficult because of the smaller window of the spleen, but can be facilitated by a more coronal approach and by paralleling the ribs (Sarwal et al., 2013). In addition to the common approaches described, several other approaches have been showed in previous studies. Among them, the posterior subcostal approach may not be practical in critically ill or mechanically ventilated patients (Fedullo et al., 1992). However, the subxiphoid approach provides another option for measuring excursion, and it is particularly useful in children (Chavhan et al., 2010). The excursion of diaphragm is usually measured during quiet breathing, deep breathing, or the sniff test.

**Table 1:** Selected studies providing direct visualization of ultrasonographic assessment of the diaphragm.

Parameters	Transducer (MHz)	Approach	Position	Condition	Subjects	References
Tdi, TFdi	10	At approximately the anterior axillary line, just cephalad to the lower costal margin	Supine	Inspiration (TLC)/end expiration	49 Healthy, 45 Stroke patients	Kim et al. (2017)
Tdi	7–13	The eighth or ninth intercostal space, anterior to the anterior axillary line	Supine	End of expiration	73 male, 77 female	Boon et al. (2013)
Tdi, TFdi	7.5–10	The eighth and ninth intercostal spaces in the right mid-axillary line	Standing	At FRC	15 Healthy	Gottesman and McCool (1997)
Tdi, TFdi, Excursion	10–14	At approximately the anterior axillary line at the 8th and 9th intercostal spaces	Supine	End deep inspiration /end-expiration	45 Stroke patients	Liu et al. (2022)
Tdi	10	In the middle of the anterior axillary and mid-axillary line of the 7th intercostal space	Supine	End-inspiration/expiration	41 Stroke patients	Kılıçoğlu et al. (2022)
Tdi	5–14	The mid-axillary lines between ribs 8 and 9 on both sides	Supine	Maximum inspiration/ end-expiration	25 Stroke patients	Cho et al. (2018)
Tdi, TFdi, Excursion	6–13 3–5.5	At the 8th–9th intercostal space of the right anterior axillary line	Supine	Calm end-expiratory/maximum end-inspiratory	60 Stroke patients	Cao et al. (2022)
Tdi, TFdi, Excursion	10–15	The zone of apposition to the rib cage, between the mid-axillary and antero-axillary line	Supine	End-inspiration/end-expiration	79 Parkinson’s disease	Yu et al. (2021)
Tdi	8–13	At approximately the anterior axillary line, just cephalad to the lower costal margin	Supine	End-expiration or functional residual capacity	50 COPD Patients	Baria et al. (2014)
Tdi, TFdi	13	The ninth or tenth intercostal, space near the mid-axillary line	Supine	End-expiratory/peak inspiratory	66 ventilated patients	Goligher et al. (2015)
Tdi	5–15	In the right intercostal space, between the antero-axillary and mid-axillary lines	Sitting	At full expiration/ inspiration	42 ALS Patients	Pinto et al. (2016)
Tdi, TFdi	6–15	In the anterior axillary line	–	End-expiration/inspiration	45 MS patients	Şahin et al. (2019)
TFdi	10–15	The ninth or tenth intercostal, space near the mid-axillary line	Supine	End-expiration/peak inspiration	122 ventilated patients	Dres et al. (2021)
TFdi	4–10	Between the mid-axillary and posterior axillary lines	Supine	End-inspiration/end-expiration	10 COPD patients	Lim et al. (2019)
Excursion	4	A low intercostal or subcostal approach using the liver or spleen as an acoustic window	Supine	Quiet breathing/deep inspiration/the sniff test	23 Healthy	Gerscovich et al. (2001)
Excursion	2.5–3.5	A low intercostal or subcostal approach using the liver or spleen as an acoustic window	Standing	Quiet breathing/deep inspiration/the sniff test	150 men, 60 women	Boussuges et al. (2009)
Excursion	–	In the longitudinal semi-coronal plane through a subcostal or intercostal approach	Supine	Spontaneous/deep respiration	23 Stroke patients	Voyvoda et al. (2012)
Excursion	1–5	A subcostal approach	Supine	Quiet and deep breathing/Voluntary sniffing	10 Stroke patients	Jung et al. (2014)
Excursion	3.5	The lower intercostal spaces in the anterior axillary lines and the liver	Supine	Quiet/deep breathing	25 COPD patients	Crimi et al. (2018)

Diaphragm thickness measurement (Table 1): With the participants in the standing, semi-recumbent, or supine position, 7–13 or 6–15 MHz linear-array transducer with B-mode or M-mode ultrasonography is used to measure the diaphragm thickness at the zone of apposition during inspiration or expiration. The probe is positioned at approximately the anterior axillary line or just cephalad to the lower costal margin at the eighth and ninth intercostal spaces. The diaphragm can be visualized as a three-layered structure consisting of a relatively non-echogenic muscular layer bounded by echogenic membranes of the peritoneum and diaphragmatic pleura with the probe perpendicular to two ribs (Supinski et al., 2018). According to the diaphragm thickness at the end-inspiration and end-expiration, the thickening fraction of the diaphragm (TFdi) can be calculated by the formula. TFdi = (end-inspiratory thickness - end-expiratory thickness)/end-expiratory thickness × 100%. Thickening fraction (TFdi) reflects on tractile activity that can be used to assess muscle function (Wait et al., 1989; Goligher et al., 2015). The ultrasound criteria of diaphragm thickness < 2.0 mm and thickening fraction (TFdi) < 20% was diagnostic of diaphragm paralysis in previous research (Gottesman and McCool, 1997).

#### 3.1.2. Extra-diaphragmatic inspiratory muscles

##### 3.1.2.1. Parasternal intercostal muscle and intercostal muscles

Together with the diaphragm, extra-diaphragmatic inspiratory muscles participate in the generation of the tidal volume (Formenti et al., 2020). The intercostal muscles can be directly visualized between the ribs (Laursen et al., 2021) and the European Respiratory Society recommends ultrasound monitoring of the intercostal muscles to assess respiratory function (Laveneziana et al., 2019). The intercostal muscles are composed of three thin layers of muscle fibers occupying each of the intercostal spaces (Formenti et al., 2020). The outer layer is external intercostal; in contrast, the layer is internal intercostal (De Troyer et al., 2005), and the inner layer is the innermost intercostal muscle (Estenne et al., 1985). The intercostal spaces contain two layers of intercostal muscle in their lateral portion but a single layer in their ventral and sometimes in their dorsal portions. Between the sternum and the chondrocostal junctions, the external intercostals are replaced by a fibrous aponeurosis, and this portion of the internal intercostals on the ventral side is conventionally called the Parasternal intercostals (De Troyer et al., 2005). The inspiratory contraction of the Parasternal intercostal muscle involves muscle shortening, acting to elevate the rib cage and expand the lung (Cala et al., 1998). Because their mass remains constant, increases in thickness can be observed using ultrasound imaging during inspiration (Vivier and Mekontso Dessap, 2020). Ultrasound has been used to investigate parasternal intercostal muscles in the easily accessible anterior parasternal region (Cala et al., 1998; Diab et al., 1998). Unlike the parasternal intercostal muscles, in the lateral and in the posterior part of the intercostal space, the internal and external intercostal muscles often overlap, making the ultrasound detection of both muscle layers impossible (Formenti et al., 2020). Therefore, ultrasonography of the intercostal muscles generally measures both the internal and external intercostal muscles. The extra-diaphragmatic respiratory muscles recruitment is a mechanism of compensation that can be activated in presence of diaphragm dysfunction (Dres et al., 2020). The parasternal intercostal muscle, as the main auxiliary inspiratory muscle, has a broad application prospect in mechanically ventilated patients, especially those with diaphragmatic dysfunction. The extra-diaphragmatic respiratory muscles recruitment is a mechanism of compensation that can be activated in presence of diaphragm dysfunction (Dres et al., 2020). The parasternal intercostal muscle, as the main auxiliary inspiratory muscle, has a broad application prospect in mechanically ventilated patients, especially those with diaphragmatic dysfunction.

Parasternal intercostal muscle thickness measurement (Table 2). Parasternal intercostal muscle ultrasound is performed with a 6–14 or 10–15 MHz linear-array transducer positioned in cranio-caudal direction at the second intercostal space, approximately 3–5 cm or 6–8 cm lateral to the sternal edge with a window visualizing between the 2nd and the 3rd rib. The supine position is usually used, and some studies used the participant at 45°. The second parasternal intercostal muscle was identified as a three-layered biconcave structure: two linear hyperechoic membranes running, respectively, from the anterior and posterior aspects of the adjoining ribs, and a medial portion with muscle echotexture. Using B-Mode or M-mode, the thickness of the parasternal intercostal muscle was measured on frozen images at end expiration and at peak inspiration during tidal breathing or total lung capacity. Change in thickness determined the thickening fraction of the parasternal intercostal muscle (TFic) as follows: TFic = (end-inspiration thickness – end-expiratory thickness)/end-expiratory thickness×100% (Dres et al., 2020, 2021). Previous research showed that a value of TFic less than 10%, associated with a TFdi greater than 20% during mechanical ventilation indicates a successful weaning trial (Formenti et al., 2020).

**Table 2:** Selected studies providing direct visualization of ultrasonographic assessment of the parasternal intercostal muscle and intercostal muscles.

Parameters	Transducer (MHz)	Approach	Position	Condition	Subjects	References
Parasternal intercostal muscle						
Tic, TFic	10–15	The level of the 2nd intercostal space, approximately 6–8 cm lateral to the sternal edge with a window visualizing the 2nd/3rd ribs	–	End-expiration/inspiration	23 Healthy, 54 mechanically ventilated patients	Dres et al. (2020)
	10–15	3 cm laterally from the sternum, and oriented along the sagittal plane, between the 2nd and the 3rd ribs	Supine	End-expiratory	50 mechanically ventilated patients	Paolo et al. (2022)
	6–14	In the sagittal plane with a window visualizing the 2nd/3rd ribs	at 45°	End-tidal expiration	32 intubated patients	Formenti et al. (2022)
	6–14	In the sagittal plane with a window visualizing the 2nd/3rd, 3rd/4th ribs	at 45°	End-tidal inspiration	20 stable COPD patients	Wallbridge et al. (2018)
Intercostal muscle						
Thickness	12	The anterior portion, 1st − 6th intercostal spaces, 25–30 mm outside from the edge of the sternum	Supine	At resting expiratory/maximal inspiratory	12 healthy men	Yoshida et al. (2019)
	12	The lateral portion, the 3rd, 6th, and 9th intercostal space, at the line connecting the axillary anterior border with the anterior superior iliac spine	Left side-lying	At resting expiratory/maximal inspiratory	12 healthy men	Yoshida et al. (2019)
	12	The posterior portion, the 3rd, 6th, and 9th intercostal spaces, and it was 50–60 mm lateral to the thoracic spinous process	Left side-lying	At resting expiratory/maximal inspiratory	12 healthy men	Yoshida et al. (2019)
	7–10	At the 8th posterior right intercostal space at medial right scapula line	Sitting	End of quiet and deep inspiration/expiration	68 older adults	Rahman et al. (2017)

Intercostal muscles thickness measurement (Table 2): Using B-mode ultrasound with 7–12 Hz linear probe to measure contractions of the intercostal muscle. The participants’ measurement used the supine position when measuring the anterior intercostal space, while the left side-lying position was used in the right lateral intercostal space and the posterior intercostal space, and the other research chose the sitting position. The measurement of anterior part of the intercostal muscle was the 1st − 6th intercostal spaces and 25–30 mm outside from the right edge of the sternum. The lateral part was the 3rd, 6th, and 9th intercostal space, at the line connecting the axillary anterior border with the anterior superior iliac spine. The posterior part was the 3rd, 6th, and 9th intercostal spaces, and it was 50–60 mm lateral to the thoracic spinous process. To measure the 8th posterior intercostal space, place the ultrasound probe at medial scapula line. Participants were in a supine position and the probe was placed at the level of the mid-axillary line and measurements were made at the 5th and 6th intercostal space (Pietton et al., 2021). One study determined the area of the intercostal muscles at maximal inhalation in one adult person and it was found that measurements at maximal inhalation were more accurate than those taken at maximal exhalation (Diab et al., 1998). Summary of the literature, previous studies have focused on the right intercostal muscles, the structure and contractile function of the bilateral intercostal muscles by ultrasound need to be further studied. Meanwhile, reference values need to be determined.

### 3.2. The scalene muscles, sternocleidomastoid muscles and the trapezius

The human inspiratory muscles in the neck include the scalene and sternomastoid. These muscles have similar respiratory actions on the chest wall and cause cranial displacement of the sternum and ribcage (Hudson et al., 2007). The human scalene are obligatory inspiratory muscles that have a greater mechanical advantage than sternomastoid. Lee et al. (2016) found that stretching of the scalene muscles improved vital capacity. Several studies used computed tomographic scan images to measure the changes in muscle length, muscle mass and size of the sternocleidomastoid muscle (Peche et al., 1996; Legrand et al., 2003).

One study evaluated the reliability of shear-wave elastography (SWE) to assess the anterior and middle scalene muscles in healthy adult subjects. Ultrasound examinations of the scalene muscles were performed by an L18–4 MHz linear-array transducer. The ultrasound transducer was placed just lateral to the thyroid lobe (Bedewi et al., 2021). However, the limitations of this study were the small sample size, and the lack of comparison with pathological tissue. Several studies used B mode linear probe of ultrasonography with frequency of 7–11 or 8.5–10.0 MHz for imaging of the trapezius (Adigozali et al., 2016; Kisilewicz et al., 2020). Subjects were asked to sit on the chair while they were in an upright and relaxed position (Adigozali et al., 2016). In order to measure the thickness and SWE of the trapezius, the position of the ultrasound probe was generally chosen the midpoint of the spinous process of 7th cervical vertebra and acromion process of right scapula were determined by palpation. However, due to the peculiarities of anatomy and the limitations of ultrasound technology, there were few ultrasound studies on the scalene, sternocleidomastoid muscles and the trapezius.

### 3.3. Expiratory muscle

The expiratory muscles include the abdominal wall muscles (transversus abdominis muscle (TrA), internal oblique muscle (IO), external oblique muscle (EO), and rectus abdominis muscle (RA)), and some of the rib cage ones (e.g., the internal intercostal muscles and the triangularis sterni muscle) (De Troyer et al., 1998; Wilson et al., 2001; De Troyer et al., 2005; De Troyer and Boriek, 2011). During tidal breathing, the expiratory muscles are largely inactive (Shi et al., 2019), although the transversus abdominis muscle may occasionally show some activity during quiet breathing (De Troyer et al., 1990). Previous study has shown that transversus abdominis has an important role in posture (Belavý et al., 2017). The contraction of the transversus abdominis muscle with the other muscles of the abdominal cavity has also been shown to increase intra-abdominal pressure (Hodges and Gandevia, 2000). Activation of the expiratory muscles during breathing occurs when the load imposed on the inspiratory muscle increases (Shi et al., 2019). In the presence of an imbalance between inspiratory muscle load and capacity, the abdominal wall muscles are recruited during expiration in a fixed hierarchy (Aliverti et al., 1997; Parthasarathy et al., 2007): initially, the transversus abdominis muscle, followed by the internal oblique muscle and the external oblique muscle, and finally the rectus abdominis muscle (Abe et al., 1996; Suzuki et al., 1999). Abdominal ultrasound allows direct visualization of the three layers of the abdominal wall muscles (Misuri et al., 1997; McMeeken et al., 2004; Rankin et al., 2006; Tahan et al., 2016). In healthy subjects, the thickness of individual abdominal wall muscles follows a certain pattern: transversus abdominis < external oblique < internal oblique < rectus abdominis (Tahan et al., 2016).

Expiratory muscle thickness measurement (Table 3): Using 7.5 or 10–15 MHz linear probe in B-mode condition positioned perpendicular to the abdominal wall. Measurements were performed with the subjects in a supine or semi-recumbent position with knees bent and the hips at 45°. To visualize the rectus abdominis muscle, the transducer is positioned in a transverse orientation approximately 2–3 cm above the umbilicus and 2–3 cm lateral from the midline. The external oblique, internal oblique, and transversus abdominis muscles can be identified as three parallel layers, usually at the anterior axillary line, midway between the inferior border of the rib cage and the iliac crest. Several studies measured rectus abdominis at 3 cm or 4 cm lateral to the umbilicus; the external oblique, internal oblique, and transverse abdominis were measured at 2.5 cm anterior to the mid-axillary line and at the midpoint between the inferior rib and iliac crest. The pressure applied to the probe should be kept to a minimum to prevent compression of the abdominal wall as this may alter the shape/thickness of the underlying muscles. Abdominal muscle thickness was performed at the end of a relaxed expiration or at end-inspiration. Thickening fraction of the expiratory abdominal muscles (TFabd) can be calculated as the magnitude of thickness increase during expiration. Change in thickness determined the thickening fraction of the expiratory abdominal muscles (TFadb) as follows: TFadb = (end-expiratory thickness − end-inspiratory thickness)/end-inspiratory thickness × 100% (Tuinman et al., 2020).

**Table 3:** Selected studies providing direct visualization of ultrasonographic assessment of the expiratory muscles’ thickness.

Parameter	Transducer (MHz)	Approach	Position	Condition	Subjects	References
IO, EO, TrA	7.5	The anterior axillary line, midway between the 12th rib and the iliac crest	Supine	End of the expiration	156, 6 healthy	Misuri et al. (1997) and Tahan et al. (2016)
IO, EO, TrA	8	2.5 cm anterior to the axillary line, at the height of the umbilicus	Supine	End of a relaxed expiration	103 healthy	Ota et al. (2012)
IO, EO, TrA	–	The middle line between the sacral crest and the inferior angle of the thoracic cage	Supine	–	24 healthy	Kang et al. (2021)
IO, EO, TrA	10	2.5 cm anterior to the mid-axillary line and at the midpoint between the inferior rib and iliac crest	Supine	End of a relaxed expiration	39, 23 healthy	Ishida et al. (2014) and Ishida et al. (2015)
IO, EO, TrA	7.5	The left side of the abdomen	Supine	The thickest muscle at end tidal	21 healthy	Sugimoto et al. (2018)
IO, EO, TrA	5–13	The mid-axillary line and the midpoint between the iliac crest and the bottom of the rib cage	Supine	End of normal expiration	21 women	Abuín-Porras et al. (2020)
IO, EO, TrA	8–12	2.5 cm anterior to the mid-axillary line at the midpoint between the inferior rib and the iliac crest	Supine	End of relaxed expiration	32, 15 stroke patients	Monjo et al. (2018) and Monjo et al. (2022)
IO, EO, TrA	5–12	At the umbilicus line and horizontally 3 cm medial to the mid-axillary line	Supine	The rest and contraction states	55 stroke patients	Kim et al. (2020)
IO, EO, TrA	7.5	Superior to the iliac crest in the transverse plane along the mid-axillary line	Crook-lying	End of expiration	33 stroke patients	Lee et al. (2018)
IO, TrA	6–12	Between the 12th rib and iliac crest 25 mm inside	Supine	At the start of expiration	23 stroke patients	Oh et al. (2016)
TrA	5–13	At the middle of the 11th costal cartilage and iliac crest, perpendicularly to the mid-axillary line	Supine	At rest (not clear)	9 stroke patients	Kelli et al. (2020)
RA	7.5	2–3 cm above the umbilicus, 2–3 cm from the midline	Supine	At the end of the expiration	156, 6 healthy	Misuri et al. (1997) and Tahan et al. (2016)
RA	8–10	4 cm lateral to the umbilicus	Supine	End of a relaxed expiration	103, 39, 23 healthy	Ota et al. (2012), Ishida et al. (2014), and Ishida et al. (2015)
RA	8–12	3 cm lateral to the umbilicus	Supine	End of relaxed expiration	32, 15 stroke patients	Monjo et al. (2018) and Monjo et al. (2022)
RA	5–12	3 cm lateral to the umbilicus	Supine	The rest and contraction states	55 stroke patients	Kim et al. (2020)
RA	7.5	2–3 cm above the umbilicus	Crook-lying	End of expiration	33 stroke patients	Lee et al. (2018)

### 3.4. Feasibility and reliability of the ultrasound measurements

To be useful, ultrasound measures should be reliable and with good reproducibility, which means that they are stable over time, have minimal variability, and have enough sensitivity to detect clinically important changes (Lexell and Downham, 2005). There were many studies on the feasibility and reliability of the diaphragmatic ultrasound measurements (Baldwin et al., 2011; Goligher et al., 2015). Ultrasonographic technique of diaphragm was previously reported to be reliable, with high intra-class correlation coefficient for intra-rater and inter-rater reliability (Gottesman and McCool, 1997; Boussuges et al., 2009). Diaphragm motion depend on the position of the subject in the study (Sarwal et al., 2013). The supine position is preferred, because there is less overall variability, less side-to-side variability, and greater reproducibility (Gerscovich et al., 2001). Baldwin et al. (2011) also found that ultrasound technique has good reliability in recumbent positions. In addition, the relationship between inspired volume and diaphragmatic motion was found to be linear (Houston et al., 1994).

On the contrary, very few studies have applied ultrasound to evaluate the extra-diaphragmatic inspiratory muscles. Parasternal intercostal muscle ultrasound may be a useful tool in the evaluation of the respiratory muscle in future studies (Tuinman et al., 2020). Dres et al. (2020) have proposed a technique to measure the parasternal intercostal muscle by ultrasonography, while their results need to be confirmed in a larger number of patients (Vivier and Mekontso Dessap, 2020). Intercostal muscle ultrasound offers a repeatable and radiation-free alternative, however requires validation. Wallbridge et al. (2018) found that the inter-rater reliability was not as strong for thickness, particularly in the third intercostal spaces bilaterally. The results of the research demonstrated that real time ultrasonography was a reliable method for measurement of upper trapezius (Adigozali et al., 2016). Other extra-diaphragmatic inspiratory muscles, such as the scalene muscles and sternocleidomastoid muscles, requires further study on their ultrasound approach, reliability, and reproducibility.

The measurement of abdominal wall muscles’ thickness was feasible in almost all healthy subjects and patients. A large number of studies evaluated the reproducibility of ultrasound imaging measures of abdominal muscle activity (McMeeken et al., 2004; English et al., 2012). Reliability in the measurement of abdominal muscle thickness was assessed in several studies and their results indicated an excellent inter-and intra-rater reproducibility in measuring the muscle thickness of these muscles (Ota et al., 2012; Shi et al., 2021).

### 3.5. Limitations of respiratory muscle ultrasonography

The operator skills, technical aspects are related to ultrasound physics, and patient characteristics, e.g., probe orientation difficulties, the small muscles, muscle edema, and the pressure of the transducer and manipulator’s hand on the wall can all affect measurements (Tuinman et al., 2020; Vivier and Mekontso Dessap, 2020). Furthermore, the spatial axial resolution of the probe plays a critical role. Comparing individual patient results should be done with a degree of caution and only after adequate training (Tuinman et al., 2020).

Previous studies have some methods to reduce variability, such as the same normalized position of the subject and the exact position of the transducer were maintained during the measurement, use the minimum amount of pressure needed to get a clear image while preventing pressure that could alter the shape or thickness of the muscle (Tuinman et al., 2020), the same observer for three different cycles of measurement, and a unified standard for measurement software, etc. (Liu et al., 2022).

### 3.6. Risk of bias

The risk of selection, performance, detection, and reporting bias in the included studies are specified in Table 4.

**Table 4:** Risk of bias.

Study	Participant selection	Evaluation protocol	Reference standard	Selective reporting
Kim et al. (2017)	●	○	○	○
Boon et al. (2013)	○	○	○	○
Gottesman and McCool (1997)	●	○	○	●
Liu et al. (2022)	●	○	○	○
Kılıçoğlu et al. (2022)	○	○	○	○
Cho et al. (2018)	○	○	○	○
Cao et al. (2022)	○	○	○	○
Yu et al. (2021)	●	○	○	○
Baria et al. (2014)	●	○	○	○
Goligher et al. (2015)	●	○	○	●
Pinto et al. (2016)	●	○	○	○
Şahin et al. (2019)	?	○	○	●
Dres et al. (2021)	●	○	○	●
Lim et al. (2019)	○	○	○	●
Gerscovich et al. (2001)	●	○	○	○
Boussuges et al. (2009)	●	○	○	●
Voyvoda et al. (2012)	●	?	○	○
Jung et al. (2014)	●	○	●	○
Crimi et al. (2018)	○	○	○	●
Dres et al. (2020)	○	○	○	●
Paolo et al. (2022)	○	○	○	●
Formenti et al. (2022)	●	○	○	○
Wallbridge et al. (2018)	●	○	○	●
Yoshida et al. (2019)	●	○	●	●
Rahman et al. (2017)	○	○	○	●
Tahan et al. (2016)	○	○	○	○
Misuri et al. (1997)	●	○	○	●
Ota et al. (2012)	○	○	○	●
Kang et al. (2021)	●	?	○	●
Ishida et al. (2015)	●	○	○	●
Ishida et al. (2014)	●	○	○	●
Sugimoto et al. (2018)	●	○	●	●
Abuín-Porras et al. (2020)	●	○	○	●
Monjo et al. (2018)	●	○	○	○
Monjo et al. (2022)	●	○	○	○
Kim et al. (2020)	○	○	●	○
Lee et al. (2018)	●	○	○	○
Oh et al. (2016)	●	○	●	●
Kelli et al. (2020)	●	○	●	○

Selection bias, participant selection; Performance bias, evaluation protocol; Detection bias, reference standard; Reporting bias, selective reporting. ○ Low risk; ● High risk; ? Unclear risk.

## 4. Clinical applications of the patients with stroke

### 4.1. Role of diaphragm ultrasound in stroke

The human diaphragm represents one of the muscles that are controlled by an automatic as well as voluntary motor system (Nakayama et al., 2004). It is thought that bilateral hemi-diaphragms are controlled by the contralateral primary motor cortex and, thus, in the presence of paralysis, the diaphragm is also affected on the same side as the paralysis (Aminoff and Sears, 1971; Cohen et al., 1994). The etiology of the paralysis is multiple (Gerscovich et al., 2001). Central nervous system disease, including brain infarction, may impair diaphragmatic motion. Because ultrasonography can distinguish a functioning from a nonfunctioning diaphragm, it can be used to diagnose both unilateral and bilateral diaphragmatic paralysis and to monitor recovery of the paralyzed diaphragm (Gottesman and McCool, 1997; Summerhill et al., 2008). Respiratory exercises could contribute to the well-being of the stroke patients and this contribution could be followed up by diaphragm ultrasound (Kılıçoğlu et al., 2022).

**Figure 2:**
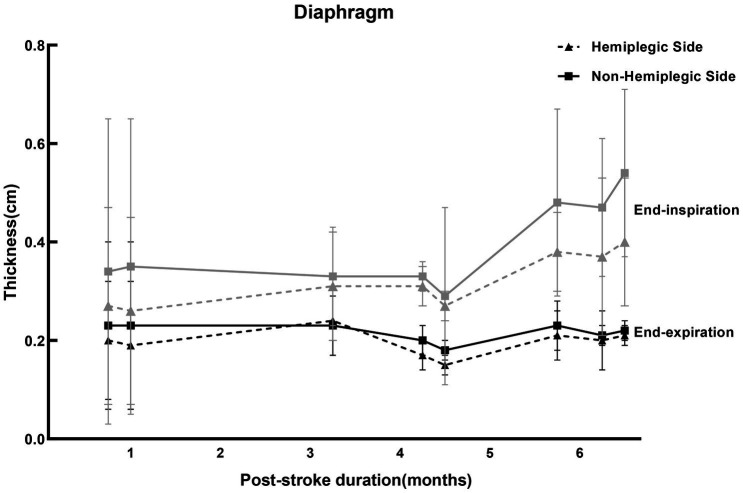
Studies for the thickness of the diaphragm ultrasound in stroke patients. The reference values of the diaphragm thickness of the stroke patients at the subacute stage at end-expiration and end-inspiration measured by ultrasound in different studies in Supplementary material 2, presented by the mean ± standard deviation. Where the gray curves (solid and dotted lines) represented the diaphragm thickness at the end of inspiration, and the black curves (solid and dotted lines) were the thickness of the diaphragm at the end of expiration. The solid lines were the thickness of the diaphragm on non-hemiplegic side, while the dotted lines were the hemiplegic side. The values on the abscissa represented the average duration of post-stroke (months).

Hemiplegic side of the diaphragm had reduced thickness and motion during voluntary inspiration on the same side of the body paralysis in patients with stroke (Laroche et al., 1988; Kim et al., 2017) and this finding was not seen during quiet breathing (Cohen et al., 1994). Our results of previous study were consistent with (Liu et al., 2022). However, there was controversy about the function of the hemiplegic side and non-hemiplegic side of the diaphragm after stroke. Some researchers showed that the motion of the diaphragm on the hemiplegic side decreases after stroke, and the contralateral showed a larger excursion compensatively (Cohen et al., 1994). However, Houston et al. (1995) found bilaterally decreased volitive diaphragmatic motion in acute cerebral infarction. Recent studies have found that the thickness and motion of the bilateral diaphragm in stroke patients were decreased (Caleffi-Pereira et al., 2018), while diaphragmatic dysfunction was more severe on the hemiplegic side (Catalá-Ripoll et al., 2020). In view of this, we reviewed the diaphragm thickness in hemiplegic patients after stroke at the subacute stage (almost within 1–6 months from the onset) with ultrasound in the literature, as shown in Figure 2. In most of the studies, the thickness of the diaphragm on the hemiplegic side of stroke patients is lower than that on the non-hemiplegic side, no matter at the end of inspiration or end of expiration (Supplementary material 2).

### 4.2. Role of expiratory muscles ultrasound in stroke

**Figure 3:**
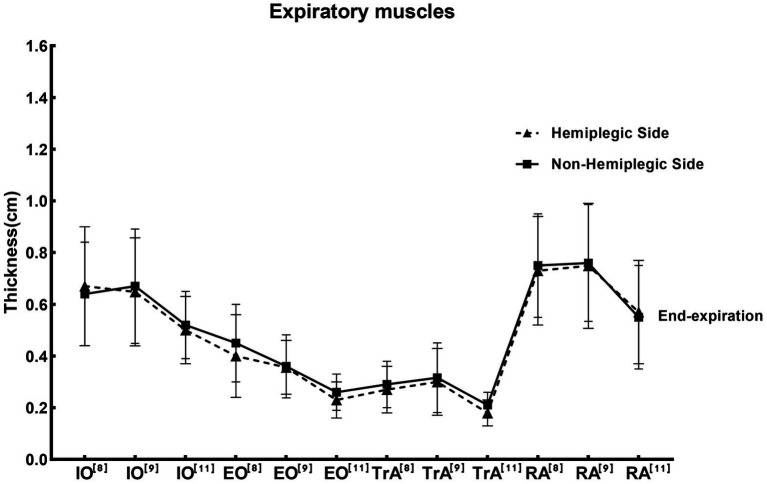
Studies for the thickness of the expiratory muscle ultrasound in stroke patients. The reference values of the thickness of expiratory muscle at end-expiration measured by ultrasound in different studies in Supplementary material 2, presented by the mean ± standard deviation. Where the solid line was the thickness of the expiratory muscles on non-hemiplegic side, while the dotted line was the hemiplegic side. Labels at the top-right of the abbreviations on the abscissa axis, such as [8],[9],[11], are shown in Supplementary material 2. The abbreviations on the abscissa represented different studies. IO, Internal oblique muscle; EO, External oblique muscle; RA, Rectus abdominis muscle; TrA, Transversus abdominis muscle.

The function of hemiplegic expiratory muscles may be affected in stroke patients. It is therefore important to determine the quantitative and qualitative changes in abdominal muscles in stroke survivors. The hemiplegic side has been found to exhibit negative changes in abdominal muscle quantity and quality compared with the non-hemiplegic side or healthy controls (Monjo et al., 2022). One study has shown that the TrA on the hemiplegic side was 16% lower in the stroke patients compared to the matched side in the healthy people (Marsden et al., 2013). However, Kelli et al. (2020) found that bilateral TrA thickness decreased in the hemiplegia patients, suggesting muscle atrophy on both sides of the trunk. Kim et al. (2020) revealed that contractility of RA and EO at the paretic side was significantly lower than at the non-paretic side, while there were no significant difference between non-paretic and paretic sides at rest. Monjo et al. (2018) indicated that changes on the hemiplegic side in stroke survivors might not occur in the abdominal muscles. Regarding the reliability of ultrasound measurements, studies have shown that ultrasound is considered a reliable method for measuring muscle thickness in acute stroke patients (English et al., 2012). We summarized the thickness of abdominal respiratory muscles in hemiplegic patients after stroke in the literature, as shown in Figure 3. In the studies included, the average post-stroke duration of the expiratory muscles-related patients with hemiplegia ranged from months to years. Due to a large duration span, there was a lack of evaluation of possible bias. Consequently, we indicated reference numbers of different studies on the abscissa replaced the average duration of post-stroke in Figure 3. For IO and RA, two of three studies indicated that the thickness of the hemiplegic side of stroke patients was lower than that of the non-hemiplegia side, and one study found the opposite conclusion. For TrA, most studies showed that the thickness on the hemiplegic side was lower than that on the non-hemiplegia side; while for EO, all three studies showed that the thickness on the hemiplegic side was lower than that on the non-hemiplegic side (Supplementary material 2).

## 5. Discussion

It is necessary to assess the muscles of respiratory to measure respiratory function. Respiratory muscle ultrasound is a widely available, highly feasible, non-invasive bedside radiation-free technique that can be easily applied in the clinic. It can provide information about the structure and function of the respiratory muscle. Moreover, ultrasonography can be used to study the contribution of the individual respiratory muscles related to pulmonary dysfunction. At present, diaphragm and expiratory muscle ultrasound has been widely used in the assessment of respiratory muscle function in healthy people, and there have been some studies on its repeatability and reliability. However, its application in diseases and the outlier values need more studies to verify. In addition, there is not enough evidence to use ultrasound measurements to assess extra-diaphragmatic inspiratory muscles, and further studies using standardized methodology are needed. The impairment of respiratory function is a frequent and serious complication for stroke patients (Kim et al., 2015). Early detection of respiratory dysfunction is important for protecting patients from comorbid pulmonary problems (Kılıçoğlu et al., 2022). By summarizing the function of respiratory muscle on hemiplegic side and non-hemiplegic side of stroke patients in the studies included, we found that the average thickness of diaphragm on the hemiplegic side of stroke patients was significantly lower than that on the non-hemiplegic side, while the average thickness of abdominal expiratory muscles on the hemiplegic side were mostly lower than that on the non-hemiplegic side. However, there are several limitations in this review. We summarized and described the applications of ultrasonic measurement of respiratory muscle according to different parameters; but there was a lack of evaluation of differences and possible bias. In addition, due to the inclusion of fewer studies, the included stroke patients were not distinguished by age, gender, type of stroke, etc., at the clinical application of this review. Due to the inconsistency of indicators reflecting diaphragmatic contractility in the included studies, diaphragmatic thickness at the end-inspiratory and end-expiratory was used instead of diaphragmatic thickening fraction in Figure 2. Moreover, the average post-stroke duration of the expiratory muscles-related patients with hemiplegia ranged from months to years in the studies included. Future research can analyze and summarize these influencing factors before elaboration.

## Author contributions

XL conceived and designed the review. XL and YY drafted the manuscript, designed the tables, and designed the figures. XL, YY, and JJ modified the language and checked the text. XL and JJ checked and confirmed the information. All authors read and approved the final manuscript.

## Funding

This research was funded by National Key Research and Development Program of the Ministry of Science and Technology of the People’s Republic of China, grant numbers 2018YFC2002300 and 2018YFC2002301.

## Conflict of interest

The authors declare that the research was conducted in the absence of any commercial or financial relationships that could be construed as a potential conflict of interest.

## Publisher’s note

All claims expressed in this article are solely those of the authors and do not necessarily represent those of their affiliated organizations, or those of the publisher, the editors and the reviewers. Any product that may be evaluated in this article, or claim that may be made by its manufacturer, is not guaranteed or endorsed by the publisher.

## References

[ref1] Abe T. Kusuhara N. Yoshimura N. Tomita T. Easton P. A. (1996). Differential respiratory activity of four abdominal muscles in humans. J. Appl. Physiol. 80, 1379–1389. doi: 10.1152/jappl.1996.80.4.1379, PMID: 8926270

[ref2] Abuín-Porras V. Maldonado-Tello P. de la Cueva-Reguera M. Rodríguez-Sanz D. Calvo-Lobo C. López-López D.. (2020). Comparison of lateral abdominal musculature activation during expiration with an expiratory flow control device versus the abdominal drawing-in maneuver in healthy women: a cross-sectional observational pilot study. Medicina 56:84. doi: 10.3390/medicina56020084, PMID: 32092978 PMC7074045

[ref3] Adigozali H. Shadmehr A. Ebrahimi E. Rezasoltani A. Naderi F. (2016). Ultrasonography for the assessment of the upper trapezius properties in healthy females: a reliability study. Muscles Ligaments Tendons J. 6, 167–172. doi: 10.11138/mltj/2016.6.1.167, PMID: 27331047 PMC4915458

[ref4] Aliverti A. Cala S. J. Duranti R. Ferrigno G. Kenyon C. M. Pedotti A.. (1997). Human respiratory muscle actions and control during exercise. J. Appl. Physiol. 83, 1256–1269. doi: 10.1152/jappl.1997.83.4.1256 9338435

[ref5] Aminoff M. J. Sears T. A. (1971). Spinal integration of segmental, cortical and breathing inputs to thoracic respiratory motoneurones. J. Physiol. 215, 557–575. doi: 10.1113/jphysiol.1971.sp009485, PMID: 4336048 PMC1331899

[ref6] Baldwin C. E. Paratz J. D. Bersten A. D. (2011). Diaphragm and peripheral muscle thickness on ultrasound: intra-rater reliability and variability of a methodology using non-standard recumbent positions. Respirology 16, 1136–1143. doi: 10.1111/j.1440-1843.2011.02005.x, PMID: 21645172

[ref7] Baria M. R. Shahgholi L. Sorenson E. J. Harper C. J. Lim K. G. Strommen J. A.. (2014). B-mode ultrasound assessment of diaphragm structure and function in patients with COPD. Chest 146, 680–685. doi: 10.1378/chest.13-2306, PMID: 24700122 PMC4151360

[ref8] Bedewi M. A. Alhariqi B. A. Aldossary N. M. Gaballah A. H. Sandougah K. J. (2021). Shear wave elastography of the scalene muscles in healthy adults. Medicine 100:e26891. doi: 10.1097/MD.0000000000026891, PMID: 34397912 PMC8360440

[ref9] Belavý D. L. Gast U. Felsenberg D. (2017). Exercise and transversus abdominis muscle atrophy after 60-d bed rest. Med. Sci. Sports Exerc. 49, 238–246. doi: 10.1249/MSS.0000000000001096, PMID: 27685010

[ref10] Benditt J. O. Boitano L. J. (2013). Pulmonary issues in patients with chronic neuromuscular disease. Am. J. Resp. Crit. Care. 187, 1046–1055. doi: 10.1164/rccm.201210-1804CI 23590262

[ref11] Boon A. J. Harper C. J. Ghahfarokhi L. S. Strommen J. A. Watson J. C. Sorenson E. J. (2013). Two-dimensional ultrasound imaging of the diaphragm: quantitative values in normal subjects. Muscle Nerve 47, 884–889. doi: 10.1002/mus.23702, PMID: 23625789

[ref12] Boussuges A. Gole Y. Blanc P. (2009). Diaphragmatic motion studied by m-mode ultrasonography: methods, reproducibility, and normal values. Chest 135, 391–400. doi: 10.1378/chest.08-1541, PMID: 19017880

[ref13] Cala S. J. Kenyon C. M. Lee A. Watkin K. Macklem P. T. Rochester D. F. (1998). Respiratory ultrasonography of human parasternal intercostal muscle in vivo. Ultrasound Med. Biol. 24, 313–326. doi: 10.1016/s0301-5629(97)00271-8, PMID: 9587987

[ref14] Caleffi-Pereira M. Pletsch-Assunção R. Cardenas L. Z. Santana P. V. Ferreira J. G. Iamonti V. C.. (2018). Unilateral diaphragm paralysis: a dysfunction restricted not just to one hemidiaphragm. BMC Pulm. Med. 18:126. doi: 10.1186/s12890-018-0698-1, PMID: 30068327 PMC6090915

[ref15] Cao H. Chen X. Ren X. Chen Z. Liu C. Ni J.. (2022). Repetitive transcranial magnetic stimulation combined with respiratory muscle training for pulmonary rehabilitation after ischemic stroke—a randomized, case-control study. Front. Aging Neurosci. 14:1006696. doi: 10.3389/fnagi.2022.1006696, PMID: 36212033 PMC9537039

[ref16] Catalá-Ripoll J. V. Monsalve-Naharro J. Á. Hernández-Fernández F. (2020). Incidence and predictive factors of diaphragmatic dysfunction in acute stroke. BMC Neurol. 20:79. doi: 10.1186/s12883-020-01664-w, PMID: 32138697 PMC7057624

[ref17] Chavhan G. B. Babyn P. S. Cohen R. A. Langer J. C. (2010). Multimodality imaging of the pediatric diaphragm: anatomy and pathologic conditions. Radiographics 30, 1797–1817. doi: 10.1148/rg.307105046, PMID: 21057121

[ref18] Cho J. E. Lee H. J. Kim M. K. Lee W. H. (2018). The improvement in respiratory function by inspiratory muscle training is due to structural muscle changes in patients with stroke: a randomized controlled pilot trial. Top. Stroke Rehabil. 25, 37–43. doi: 10.1080/10749357.2017.1383681, PMID: 29061084

[ref19] Cohen E. Mier A. Heywood P. Murphy K. Boultbee J. Guz A. (1994). Diaphragmatic movement in hemiplegic patients measured by ultrasonography. Thorax 49, 890–895. doi: 10.1136/thx.49.9.890, PMID: 7940429 PMC475186

[ref20] Crimi C. Heffler E. Augelletti T. Campisi R. Noto A. Vancheri C.. (2018). Utility of ultrasound assessment of diaphragmatic function before and after pulmonary rehabilitation in COPD patients. Int. J. Chron. Obstruct. Pulmon. Dis. 13, 3131–3139. doi: 10.2147/COPD.S171134, PMID: 30349221 PMC6183592

[ref21] De Troyer A. Boriek A. M. (2011). Mechanics of the respiratory muscles. Compr. Physiol. 1, 1273–1300. doi: 10.1002/cphy.c100009 23733642

[ref22] De Troyer A. Estenne M. Ninane V. Van Gansbeke D. Gorini M. (1990). Transversus abdominis muscle function in humans. J. Appl. Physiol. 68, 1010–1016. doi: 10.1152/jappl.1990.68.3.1010 2140344

[ref23] De Troyer A. Kirkwood P. A. Wilson T. A. (2005). Respiratory action of the intercostal muscles. Physiol. Rev. 85, 717–756. doi: 10.1152/physrev.00007.2004 15788709

[ref24] De Troyer A. Legrand A. Gevenois P. A. Wilson T. A. (1998). Mechanical advantage of the human parasternal intercostal and triangularis sterni muscles. J. Physiol. 513, 915–925. doi: 10.1111/j.1469-7793.1998.915ba.x, PMID: 9824728 PMC2231324

[ref25] Diab K. M. Shalabi A. Sevastik J. A. Guntner P. (1998). A method for morphometric study of the intercostal muscles by high-resolution ultrasound. Eur. Spine J. 7, 224–228. doi: 10.1007/s005860050061, PMID: 9684956 PMC3611245

[ref26] Dres M. Dubé B. Goligher E. Vorona S. Demiri S. Morawiec E.. (2020). Usefulness of parasternal intercostal muscle ultrasound during weaning from mechanical ventilation. Anesthesiology 132, 1114–1125. doi: 10.1097/ALN.0000000000003191, PMID: 32084029

[ref27] Dres M. Similowski T. Goligher E. C. Pham T. Sergenyuk L. Telias I.. (2021). Dyspnoea and respiratory muscle ultrasound to predict extubation failure. Eur. Respir. J. 58:2100002. doi: 10.1183/13993003.00002-2021, PMID: 33875492

[ref28] English C. K. Thoirs K. A. Fisher L. McLennan H. Bernhardt J. (2012). Ultrasound is a reliable measure of muscle thickness in acute stroke patients, for some, but not all anatomical sites: a study of the intra-rater reliability of muscle thickness measures in acute stroke patients. Ultrasound Med. Biol. 38, 368–376. doi: 10.1016/j.ultrasmedbio.2011.12.012, PMID: 22266233

[ref29] Estenne M. Yernault J. C. De Troyer A. (1985). Rib cage and diaphragm-abdomen compliance in humans: effects of age and posture. J. Appl. Physiol. 59, 1842–1848. doi: 10.1152/jappl.1985.59.6.1842, PMID: 4077793

[ref30] Fedullo A. J. Lerner R. M. Gibson J. Shayne D. S. (1992). Sonographic measurement of diaphragmatic motion after coronary artery bypass surgery. Chest 102, 1683–1686. doi: 10.1378/chest.102.6.1683, PMID: 1359958

[ref31] Formenti P. Umbrello M. Castagna V. Cenci S. Bichi F. Pozzi T.. (2022). Respiratory and peripheral muscular ultrasound characteristics in ICU COVID 19 ARDS patients. J. Crit. Care 67, 14–20. doi: 10.1016/j.jcrc.2021.09.007, PMID: 34600218 PMC8480969

[ref32] Formenti P. Umbrello M. Dres M. Chiumello D. (2020). Ultrasonographic assessment of parasternal intercostal muscles during mechanical ventilation. Ann. Intensive Care 10:120. doi: 10.1186/s13613-020-00735-y, PMID: 32894372 PMC7475948

[ref33] Gerscovich E. O. Cronan M. McGahan J. P. Jain K. Jones C. D. McDonald C. (2001). Ultrasonographic evaluation of diaphragmatic motion. J. Ultrasound Med. 20, 597–604. doi: 10.7863/jum.2001.20.6.597, PMID: 11400933

[ref34] Goligher E. C. Laghi F. Detsky M. E. Farias P. Murray A. Brace D.. (2015). Measuring diaphragm thickness with ultrasound in mechanically ventilated patients: feasibility, reproducibility and validity. Intens. Care Med. 41, 642–649. doi: 10.1007/s00134-015-3687-3, PMID: 25693448

[ref35] Gottesman E. McCool F. D. (1997). Ultrasound evaluation of the paralyzed diaphragm. Am. J. Resp. Crit. Care. 155, 1570–1574. doi: 10.1164/ajrccm.155.5.9154859, PMID: 9154859

[ref36] Hodges P. W. Gandevia S. C. (2000). Changes in intra-abdominal pressure during postural and respiratory activation of the human diaphragm. J. Appl. Physiol. 89, 967–976. doi: 10.1152/jappl.2000.89.3.967, PMID: 10956340

[ref37] Houston J. G. Angus R. M. Cowan M. D. McMillan N. C. Thomson N. C. (1994). Ultrasound assessment of normal hemidiaphragmatic movement: relation to inspiratory volume. Thorax 49, 500–503. doi: 10.1136/thx.49.5.500, PMID: 8016774 PMC474874

[ref38] Houston J. G. Morris A. D. Grosset D. G. Lees K. R. McMillan N. Bone I. (1995). Ultrasonic evaluation of movement of the diaphragm after acute cerebral infarction. J. Neurol. Neurosurg. Psychiatry 58, 738–741. doi: 10.1136/jnnp.58.6.738, PMID: 7608679 PMC1073558

[ref39] Hudson A. L. Gandevia S. C. Butler J. E. (2007). The effect of lung volume on the co-ordinated recruitment of scalene and sternomastoid muscles in humans. J. Physiol. 584, 261–270. doi: 10.1113/jphysiol.2007.137240, PMID: 17690147 PMC2277075

[ref41] Ishida H. Kobara K. Osaka H. Suehiro T. Ito T. Kurozumi C.. (2014). Correlation between peak expiratory flow and abdominal muscle thickness. J. Phys. Ther. Sci. 26, 1791–1793. doi: 10.1589/jpts.26.1791, PMID: 25435702 PMC4242957

[ref42] Ishida H. Suehiro T. Kurozumi C. Ono K. Watanabe S. (2015). Correlation between abdominal muscle thickness and maximal expiratory pressure. J. Ultras. Med. 34, 2001–2005. doi: 10.7863/ultra.14.12006, PMID: 26396169

[ref43] Jandt S. R. Da Sil Caballero R. M. Junior L. A. F. Dias A. S. (2011). Correlation between trunk control, respiratory muscle strength and spirometry in patients with stroke: an observational study. Physiother. Res. Int. 16, 218–224. doi: 10.1002/pri.495, PMID: 21157882

[ref44] Jung K. Park J. Hwang D. Kim J. Kim J. (2014). Ultrasonographic diaphragmatic motion analysis and its correlation with pulmonary function in hemiplegic stroke patients. Ann. Rehabil. Med. 38, 29–37. doi: 10.5535/arm.2014.38.1.29, PMID: 24639923 PMC3953360

[ref45] Kang K. W. Kwon Y. H. Son S. M. (2021). Ultrasound measurement of the transverse abdominis, internal oblique, and external oblique muscles associated with forward head posture and reduced Cranio-vertebral angle. Med. Sci. Monitor. 27:e928987. doi: 10.12659/MSM.928987, PMID: 34097670 PMC8194289

[ref46] Kelli A. Kellis E. Galanis N. Dafkou K. Sahinis C. Ellinoudis A. (2020). Transversus abdominis thickness at rest and exercise in individuals with poststroke hemiparesis. Sports 8:86. doi: 10.3390/sports8060086, PMID: 32545550 PMC7353629

[ref47] Kılıçoğlu M. S. Yurdakul O. V. Çelik Y. Aydın T. (2022). Investigating the correlation between pulmonary function tests and ultrasonographic diaphragm measurements and the effects of respiratory exercises on these parameters in hemiplegic patients. Top. Stroke Rehabil. 29, 218–229. doi: 10.1080/10749357.2021.1911748, PMID: 33844946

[ref001] Kim C. Lee J. Kim H. Kim I. (2015). Effects of the combination of respiratory muscle training and abdominal drawing-in maneuver on respiratory muscle activity in patients with post-stroke hemiplegia: A pilot randomized controlled trial. Top. Stroke Rehabil. 22, 262–270. doi: 10.1179/1074935714Z.0000000020 26258451

[ref48] Kim Y. Kim J. Nam H. Kim H. D. Eom M. J. Jung S. H.. (2020). Ultrasound imaging of the trunk muscles in acute stroke patients and relations with balance scales. Ann. Rehabil. Med. 44, 273–283. doi: 10.5535/arm.19125, PMID: 32721990 PMC7463119

[ref49] Kim M. Lee K. Cho J. Lee W. (2017). Diaphragm thickness and inspiratory muscle functions in chronic stroke patients. Med. Sci. Monitor. 23, 1247–1253. doi: 10.12659/MSM.900529, PMID: 28284044 PMC5358861

[ref50] Kisilewicz A. Madeleine P. Ignasiak Z. Ciszek B. Kawczynski A. Larsen R. G. (2020). Eccentric exercise reduces upper trapezius muscle stiffness assessed by shear wave elastography and myotonometry. Front. Bioeng. Biotechnol. 8:928. doi: 10.3389/fbioe.2020.00928, PMID: 32903634 PMC7438744

[ref51] Laroche C. M. Mier A. K. Moxham J. Green M. (1988). Diaphragm strength in patients with recent hemidiaphragm paralysis. Thorax 43, 170–174. doi: 10.1136/thx.43.3.170, PMID: 3261460 PMC461156

[ref52] Laursen C. B. Clive A. Hallifax R. Pietersen P. I. Asciak R. Davidsen J. R. M.. (2021). European respiratory society statement on thoracic ultrasound. Eur. Respir. J. 57:2001519. doi: 10.1183/13993003.01519-2020, PMID: 33033148

[ref53] Laveneziana P. Albuquerque A. Aliverti A. Babb T. Barreiro E. Dres M.. (2019). ERS statement on respiratory muscle testing at rest and during exercise. Eur. Respir. J. 53:1801214. doi: 10.1183/13993003.01214-2018, PMID: 30956204

[ref54] Lee K. Cho J. Hwang D. Lee W. (2018). Decreased respiratory muscle function is associated with impaired trunk balance among chronic stroke patients: a cross-sectional study. Tohoku J. Exp. Med. 245, 79–88. doi: 10.1620/tjem.245.79, PMID: 29848898

[ref55] Lee J. Hwang S. Han S. Han D. (2016). Effects of stretching the scalene muscles on slow vital capacity. J. Phys. Ther. Sci. 28, 1825–1828. doi: 10.1589/jpts.28.1825, PMID: 27390425 PMC4932066

[ref57] Legrand A. Schneider E. Gevenois P. De Troyer A. (2003). Respiratory effects of the scalene and sternomastoid muscles in humans. J. Appl. Physiol. 94, 1467–1472. doi: 10.1152/japplphysiol.00869.2002, PMID: 12626472

[ref58] Lexell J. E. Downham D. Y. (2005). How to assess the reliability of measurements in rehabilitation [retracted]. Am. J. Phys. Med. Rehab. 84, 719–723. doi: 10.1097/01.phm.0000176452.17771.20 16141752

[ref59] Lim S. Y. Lim G. Lee Y. J. Cho Y. J. Park J. S. Yoon H. I.. (2019). Ultrasound assessment of diaphragmatic function during acute exacerbation of chronic obstructive pulmonary disease: a pilot study. Int. J. Chron. Obstruct. Pulmon. Dis. 14, 2479–2484. doi: 10.2147/COPD.S214716, PMID: 31806957 PMC6844220

[ref60] Liu X. Qu Q. Deng P. Zhao Y. Liu C. Fu C.. (2022). Assessment of diaphragm in hemiplegic patients after stroke with ultrasound and its correlation of extremity motor and balance function. Brain Sci. 12:882. doi: 10.3390/brainsci12070882, PMID: 35884689 PMC9313444

[ref61] Marsden J. F. Hough A. Shum G. Shaw S. Freeman J. A. (2013). Deep abdominal muscle activity following supratentorial stroke. J. Electromyogr. Kines. 23, 985–990. doi: 10.1016/j.jelekin.2013.04.003, PMID: 23684056

[ref62] Matamis D. Soilemezi E. Tsagourias M. Akoumianaki E. Dimassi S. Boroli F.. (2013). Sonographic evaluation of the diaphragm in critically ill patients. Technique and clinical applications. Intensive Care Med. 39, 801–810. doi: 10.1007/s00134-013-2823-1, PMID: 23344830

[ref63] McMeeken J. M. Beith I. D. Newham D. J. Milligan P. Critchley D. J. (2004). The relationship between EMG and change in thickness of transversus abdominis. Clin. Biomech. 19, 337–342. doi: 10.1016/j.clinbiomech.2004.01.007, PMID: 15109752

[ref64] Misuri G. Colagrande S. Gorini M. Iandelli I. Mancini M. Duranti R.. (1997). In vivo ultrasound assessment of respiratory function of abdominal muscles in normal subjects. Eur. Respir. J. 10, 2861–2867. doi: 10.1183/09031936.97.10122861, PMID: 9493674

[ref65] Monjo H. Fukumoto Y. Asai T. Ohshima K. Kubo H. Tajitsu H.. (2022). Changes in muscle thickness and echo intensity in chronic stroke survivors: a 2-year longitudinal study. J. Clin. Neurol. 18, 308–314. doi: 10.3988/jcn.2022.18.3.308, PMID: 35196746 PMC9163946

[ref66] Monjo H. Fukumoto Y. Asai T. Shuntoh H. (2018). Muscle thickness and echo intensity of the abdominal and lower extremity muscles in stroke survivors. J. Clin. Neurol. 14, 549–554. doi: 10.3988/jcn.2018.14.4.549, PMID: 30198230 PMC6172490

[ref67] Nakayama T. Fujii Y. Suzuki K. Kanazawa I. Nakada T. (2004). The primary motor area for voluntary diaphragmatic motion identified by high field fMRI. J. Neurol. 251, 730–735. doi: 10.1007/s00415-004-0413-4, PMID: 15311350

[ref68] Oh D. Kim G. Lee W. Shin M. M. (2016). Effects of inspiratory muscle training on balance ability and abdominal muscle thickness in chronic stroke patients. J. Phys. Ther. Sci. 28, 107–111. doi: 10.1589/jpts.28.107, PMID: 26957739 PMC4755985

[ref69] Ota M. Ikezoe T. Kaneoka K. Ichihashi N. (2012). Age-related changes in the thickness of the deep and superficial abdominal muscles in women. Arch. Gerontol. Geriat. 55, e26–e30. doi: 10.1016/j.archger.2012.03.007, PMID: 22483589

[ref70] Paolo F. Valentina D. G. Silvia C. Tommaso P. Elena C. Martin D.. (2022). The possible predictive value of muscle ultrasound in the diagnosis of ICUAW in long-term critically ill patients. J. Crit. Care 71:154104. doi: 10.1016/j.jcrc.2022.154104, PMID: 35797827

[ref71] Parthasarathy S. Jubran A. Laghi F. Tobin M. J. (2007). Sternomastoid, rib cage, and expiratory muscle activity during weaning failure. J. Appl. Physiol. 103, 140–147. doi: 10.1152/japplphysiol.00904.2006, PMID: 17395760

[ref72] Peche R. Estenne M. Gevenois P. A. Brassinne E. Yernault J. C. De Troyer A. (1996). Sternomastoid muscle size and strength in patients with severe chronic obstructive pulmonary disease. Am. J. Resp. Crit. Care. 153, 422–425. doi: 10.1164/ajrccm.153.1.8542153, PMID: 8542153

[ref73] Pietton R. David M. Hisaund A. Langlais T. Skalli W. Vialle R.. (2021). Biomechanical evaluation of intercostal muscles in healthy children and adolescent idiopathic scoliosis: a preliminary study. Ultrasound Med. Biol. 47, 51–57. doi: 10.1016/j.ultrasmedbio.2020.09.011, PMID: 33077337

[ref74] Pinto S. Alves P. Pimentel B. Swash M. de Carvalho M. (2016). Ultrasound for assessment of diaphragm in ALS. Clin. Neurophysiol. 127, 892–897. doi: 10.1016/j.clinph.2015.03.024, PMID: 25971723

[ref75] Rahman N. N. Singh D. Lee R. Y. (2017). Correlation between thoracolumbar curvatures and respiratory function in older adults. Clin. Interv. Aging 12, 523–529. doi: 10.2147/CIA.S110329, PMID: 28352165 PMC5358964

[ref76] Rankin G. Stokes M. Newham D. J. (2006). Abdominal muscle size and symmetry in normal subjects. Muscle Nerve 34, 320–326. doi: 10.1002/mus.20589, PMID: 16775833

[ref77] Rochester C. L. Mohsenin V. (2002). Respiratory complications of stroke. Semin. Respir. Crit. Care Med. 23, 248–260. doi: 10.1055/s-2002-33033 16088617

[ref78] Şahin H. Doğan A. Ekiz T. (2019). Ultrasonographic evaluation of the diaphragm thickness in patients with multiple sclerosis. Mult. Scler. Relat. Disord. 36:101369. doi: 10.1016/j.msard.2019.08.011, PMID: 31446243

[ref79] Sarwal A. Walker F. O. Cartwright M. S. (2013). Neuromuscular ultrasound for evaluation of the diaphragm. Muscle Nerve 47, 319–329. doi: 10.1002/mus.23671, PMID: 23382111 PMC3581727

[ref81] Sferrazza Papa G. F. Pellegrino G. M. Di Marco F. Imeri G. Brochard L. Goligher E.. (2016). A review of the ultrasound assessment of diaphragmatic function in clinical practice. Respiration 91, 403–411. doi: 10.1159/000446518, PMID: 27216909

[ref82] Shi Z. de Vries H. de Grooth H. Jonkman A. H. Zhang Y. Haaksma M.. (2021). Changes in respiratory muscle thickness during mechanical ventilation: focus on expiratory muscles. Anesthesiology 134, 748–759. doi: 10.1097/ALN.0000000000003736, PMID: 33711154

[ref83] Shi Z. Jonkman A. de Vries H. Jansen D. Ottenheijm C. Girbes A.. (2019). Expiratory muscle dysfunction in critically ill patients: towards improved understanding. Intens. Care Med. 45, 1061–1071. doi: 10.1007/s00134-019-05664-4, PMID: 31236639 PMC6667683

[ref84] Sugimoto T. Yokogawa M. Miaki H. Madokoro S. Nakagawa T. (2018). Changes in thickness of the transversus abdominis during the abdominal drawing-in manoeuvre and expiratory muscle training in elderly people. J. Phys. Ther. Sci. 30, 119–123. doi: 10.1589/jpts.30.119, PMID: 29410580 PMC5788789

[ref85] Summerhill E. M. El-Sameed Y. A. Glidden T. J. McCool F. D. (2008). Monitoring recovery from diaphragm paralysis with ultrasound. Chest 133, 737–743. doi: 10.1378/chest.07-2200, PMID: 18198248

[ref86] Supinski G. S. Morris P. E. Dhar S. Callahan L. A. (2018). Diaphragm dysfunction in critical illness. Chest 153, 1040–1051. doi: 10.1016/j.chest.2017.08.1157, PMID: 28887062 PMC6026291

[ref87] Suzuki J. Tanaka R. Yan S. Chen R. Macklem P. T. Kayser B. (1999). Assessment of abdominal muscle contractility, strength, and fatigue. Am. J. Respir. Crit. Care Med. 159, 1052–1060. doi: 10.1164/ajrccm.159.4.9803025, PMID: 10194145

[ref88] Tahan N. Khademi-Kalantari K. Mohseni-Bandpei M. A. Mikaili S. Baghban A. A. Jaberzadeh S. (2016). Measurement of superficial and deep abdominal muscle thickness: an ultrasonography study. J. Physiol. Anthropol. 35:17. doi: 10.1186/s40101-016-0106-6, PMID: 27553830 PMC4995748

[ref89] Tuinman P. R. Jonkman A. H. Dres M. Shi Z. Goligher E. C. Goffi A.. (2020). Respiratory muscle ultrasonography: methodology, basic and advanced principles and clinical applications in ICU and ED patients—a narrative review. Intens. Care Med. 46, 594–605. doi: 10.1007/s00134-019-05892-8, PMID: 31938825 PMC7103016

[ref90] Ueki J. De Bruin P. F. Pride N. B. (1995). In vivo assessment of diaphragm contraction by ultrasound in normal subjects. Thorax 50, 1157–1161. doi: 10.1136/thx.50.11.1157, PMID: 8553271 PMC475087

[ref91] Vivier E. Mekontso Dessap A. (2020). Bedside ultrasound for weaning from mechanical ventilation. Anesthesiology 132, 947–948. doi: 10.1097/ALN.0000000000003235 32265347

[ref92] Voyvoda N. Yücel C. Karatas G. Oguzulgen I. Oktar S. (2012). An evaluation of diaphragmatic movements in hemiplegic patients. Brit. J. Radiol. 85, 411–414. doi: 10.1259/bjr/71968119, PMID: 21712430 PMC3485549

[ref93] Wait J. L. Nahormek P. A. Yost W. T. Rochester D. P. (1989). Diaphragmatic thickness-lung volume relationship in vivo. J. Appl. Physiol. 67, 1560–1568. doi: 10.1152/jappl.1989.67.4.1560, PMID: 2676955

[ref94] Wallbridge P. Parry S. M. Das S. Law C. Hammerschlag G. Irving L.. (2018). Parasternal intercostal muscle ultrasound in chronic obstructive pulmonary disease correlates with spirometric severity. Sci. Rep. 8:15274. doi: 10.1038/s41598-018-33666-7, PMID: 30323179 PMC6189142

[ref95] Wilson T. A. Legrand A. Gevenois P. A. Troyer A. (2001). Respiratory effects of the external and internal intercostal muscles in humans. J. Physiol. 530, 319–330. doi: 10.1111/j.1469-7793.2001.0319l.x, PMID: 11208979 PMC2278403

[ref96] Yoshida R. Tomita K. Kawamura K. Nozaki T. Setaka Y. Monma M.. (2019). Measurement of intercostal muscle thickness with ultrasound imaging during maximal breathing. J. Phys. Ther. Sci. 31, 340–343. doi: 10.1589/jpts.31.340, PMID: 31037006 PMC6451941

[ref97] Yu X. Jiang H. Zhang C. Jin Z. Gao L. Wang R.. (2021). The role of the diaphragm in postural stability and visceral function in Parkinson’s disease. Front. Aging Neurosci. 13:785020. doi: 10.3389/fnagi.2021.785020, PMID: 35002681 PMC8733584

