# Supplementary material for: Respiratory muscle ultrasonography evaluation and its clinical application in stroke patients: A review
Source: Front Neurosci. 2023 Apr 6;17:1132335. doi: 10.3389/fnins.2023.1132335 (PMC10115993; doi:10.3389/fnins.2023.1132335)
Supplement: Supplementary file 1 [file Data_Sheet_1.docx]

**MEDLINE**

Ultrasonography, Mammary/ or Ultrasonography, Doppler, Duplex/ or Ultrasonography, Prenatal/ or Ultrasonography, Doppler, Color/ or Ultrasonography, Doppler, Pulsed/ or Ultrasonography, Doppler/ or Ultrasonography, Doppler, Transcranial/ or Ultrasonography/ or Ultrasonography, Interventional/

Diaphragm/ or Respiratory Muscles/ or Intercostal Muscles/ or the trapezius/ or the sternocleidomastoid muscles/ or the scalene muscles/ or parasternal intercostal muscle/ or inspiratory muscle/ or expiratory muscle/

Stroke Rehabilitation/ or Ischemic Stroke/ or Stroke Volume/ or Thrombotic Stroke/ or Hemorrhagic Stroke/ or "National Institute of Neurological Disorders and Stroke (U.S.)"/ or Heat Stroke/ or Stroke/ or Embolic Stroke/ or Stroke, Lacunar/

# Ultrasonography[MeSH/Abstracts]:Ultrasonography, Mammary/ or Ultrasonography, Doppler, Duplex/ or Ultrasonography, Prenatal/ or Ultrasonography, Doppler, Color/ or Ultrasonography, Doppler, Pulsed/ or Ultrasonography, Doppler/ or Ultrasonography, Doppler, Transcranial/ or Ultrasonography/ or Ultrasonography, Interventional/

# Diaphragm/[Title/Abstract]

# Respiratory Muscles/[Title/Abstract]

# Intercostal Muscles/[Title/Abstract]

# the trapezius/[Title/Abstract]

# the sternocleidomastoid muscles/[Title/Abstract]

# the scalene muscles/[Title/Abstract]

# parasternal intercostal muscle/[Title/Abstract]

# inspiratory muscle/[Title/Abstract]

# expiratory muscle/[Title/Abstract]

# #2 or #3 or #4 or #5 or #6 or #7 or #8 or #9 or #10

# #1 and #11

# stroke[MeSH/Abstracts]: Stroke Rehabilitation/ or Ischemic Stroke/ or Stroke Volume/ or Thrombotic Stroke/ or Hemorrhagic Stroke/ or "National Institute of Neurological Disorders and Stroke (U.S.)"/ or Heat Stroke/ or Stroke/ or Embolic Stroke/ or Stroke, Lacunar/

# #1 and #11 and #13

# [Web of Science](http://www.lib.wh.sdu.edu.cn/dn/navigation175.html)

# TS=(Ultrasonography)

# ((((((((TS=(Diaphragm)) OR TS=(Respiratory Muscles)) OR TS=(Intercostal Muscles)) OR TS=(the trapezius)) OR TS=(the sternocleidomastoid muscles)) OR TS=(the scalene muscles)) OR TS=(parasternal intercostal muscle)) OR TS=(inspiratory muscle)) OR TS=(expiratory muscle)

# #1 AND #2

# TS=(stroke)

# #1 AND #2 AND #4

# Pubmed

# Ultrasonography

- Diagnostic Ultrasound
- Diagnostic Ultrasounds
- Ultrasound, Diagnostic
- Ultrasounds, Diagnostic
- Ultrasound Imaging
- Imaging, Ultrasound
- Imagings, Ultrasound
- Echotomography
- Ultrasonic Imaging
- Imaging, Ultrasonic
- Sonography, Medical
- Medical Sonography
- Ultrasonographic Imaging
- Imaging, Ultrasonographic
- Imagings, Ultrasonographic
- Ultrasonographic Imagings
- Echography
- Diagnosis, Ultrasonic
- Diagnoses, Ultrasonic
- Ultrasonic Diagnoses
- Ultrasonic Diagnosis
- Echotomography, Computer
- Computer Echotomography
- Tomography, Ultrasonic
- Ultrasonic Tomography

"Ultrasonography"[Title/Abstract] OR "diagnostic ultrasound"[Title/Abstract] OR "diagnostic ultrasounds"[Title/Abstract] OR "ultrasound diagnostic"[Title/Abstract] OR "ultrasounds diagnostic"[Title/Abstract] OR "ultrasound imaging"[Title/Abstract] OR "imaging ultrasound"[Title/Abstract] OR (("image"[All Fields] OR "image s"[All Fields] OR "imaged"[All Fields] OR "imager"[All Fields] OR "imager s"[All Fields] OR "imagers"[All Fields] OR "images"[All Fields] OR "Imaging"[All Fields] OR "imaging s"[All Fields] OR "Imagings"[All Fields]) AND "Ultrasound"[Title/Abstract]) OR "Echotomography"[Title/Abstract] OR "ultrasonic imaging"[Title/Abstract] OR "imaging ultrasonic"[Title/Abstract] OR "sonography medical"[Title/Abstract] OR "medical sonography"[Title/Abstract] OR "ultrasonographic imaging"[Title/Abstract] OR "imaging ultrasonographic"[Title/Abstract] OR (("image"[All Fields] OR "image s"[All Fields] OR "imaged"[All Fields] OR "imager"[All Fields] OR "imager s"[All Fields] OR "imagers"[All Fields] OR "images"[All Fields] OR "Imaging"[All Fields] OR "imaging s"[All Fields] OR "Imagings"[All Fields]) AND "Ultrasonographic"[Title/Abstract]) OR "ultrasonographic imagings"[Title/Abstract] OR "Echography"[Title/Abstract] OR "diagnosis ultrasonic"[Title/Abstract] OR "diagnoses ultrasonic"[Title/Abstract] OR "ultrasonic diagnoses"[Title/Abstract] OR "ultrasonic diagnosis"[Title/Abstract] OR "echotomography computer"[Title/Abstract] OR "computer echotomography"[Title/Abstract] OR "tomography ultrasonic"[Title/Abstract] OR "ultrasonic tomography"[Title/Abstract]

Diaphragm

- Diaphragms
- Respiratory Diaphragm
- Diaphragm, Respiratory
- Diaphragms, Respiratory
- Respiratory Diaphragms

"Diaphragm"[Title/Abstract] OR "Diaphragms"[Title/Abstract] OR "respiratory diaphragm"[Title/Abstract] OR "diaphragm respiratory"[Title/Abstract] OR (("Diaphragm"[MeSH Terms] OR "Diaphragm"[All Fields] OR "contraceptive devices, female"[MeSH Terms] OR ("contraceptive"[All Fields] AND "devices"[All Fields] AND "female"[All Fields]) OR "female contraceptive devices"[All Fields] OR "Diaphragms"[All Fields] OR "diaphragm s"[All Fields] OR "diaphragmal"[All Fields]) AND "Respiratory"[Title/Abstract]) OR (("eur med j respir"[Journal] OR "Respiratory"[All Fields]) AND "Diaphragms"[Title/Abstract])

# Respiratory Muscles

- Muscle, Respiratory
- Muscles, Respiratory
- Respiratory Muscle
- Ventilatory Muscles
- Muscle, Ventilatory
- Muscles, Ventilatory
- Ventilatory Muscle

"respiratory muscles"[Title/Abstract] OR "muscle respiratory"[Title/Abstract] OR "muscles respiratory"[Title/Abstract] OR "respiratory muscle"[Title/Abstract] OR "ventilatory muscles"[Title/Abstract] OR "muscle ventilatory"[Title/Abstract] OR "muscles ventilatory"[Title/Abstract] OR "ventilatory muscle"[Title/Abstract]

# Intercostal Muscles

- Intercostal Muscle
- Muscle, Intercostal
- Muscles, Intercostal

"intercostal muscles"[Title/Abstract] OR "intercostal muscle"[Title/Abstract] OR "muscle intercostal"[Title/Abstract] OR "muscles intercostal"[Title/Abstract]

# trapezius

# sternocleidomastoid muscles

# scalene muscles

# parasternal intercostal muscle

# inspiratory muscle

# expiratory muscle

# Stroke

- Strokes
- Cerebrovascular Accident
- Cerebrovascular Accidents
- CVA (Cerebrovascular Accident)
- CVAs (Cerebrovascular Accident)
- Cerebrovascular Apoplexy
- Apoplexy, Cerebrovascular
- Vascular Accident, Brain
- Brain Vascular Accident
- Brain Vascular Accidents
- Vascular Accidents, Brain
- Cerebrovascular Stroke
- Cerebrovascular Strokes
- Stroke, Cerebrovascular
- Strokes, Cerebrovascular
- Apoplexy
- Cerebral Stroke
- Cerebral Strokes
- Stroke, Cerebral
- Strokes, Cerebral
- Stroke, Acute
- Acute Stroke
- Acute Strokes
- Strokes, Acute
- Cerebrovascular Accident, Acute
- Acute Cerebrovascular Accident
- Acute Cerebrovascular Accidents
- Cerebrovascular Accidents, Acute

# Hemiplegia

- Hemiplegias
- Hemiplegia, Transient
- Hemiplegias, Transient
- Transient Hemiplegia
- Transient Hemiplegias
- Monoplegia
- Monoplegias
- Hemiplegia, Post-Ictal
- Hemiplegia, Post Ictal
- Hemiplegias, Post-Ictal
- Post-Ictal Hemiplegia
- Post-Ictal Hemiplegias
- Hemiplegia, Crossed
- Crossed Hemiplegia
- Crossed Hemiplegias
- Hemiplegias, Crossed
- Hemiplegia, Flaccid
- Flaccid Hemiplegia
- Flaccid Hemiplegias
- Hemiplegias, Flaccid
- Hemiplegia, Infantile
- Hemiplegias, Infantile
- Infantile Hemiplegia
- Infantile Hemiplegias
- Hemiplegia, Spastic
- Hemiplegias, Spastic
- Spastic Hemiplegia
- Spastic Hemiplegias

"Stroke"[Title/Abstract] OR "Strokes"[Title/Abstract] OR "cerebrovascular accident"[Title/Abstract] OR "cerebrovascular accidents"[Title/Abstract] OR (("Stroke"[MeSH Terms] OR "Stroke"[All Fields] OR "cva"[All Fields]) AND "cerebrovascular accident"[Title/Abstract]) OR ("CVAs"[All Fields] AND "cerebrovascular accident"[Title/Abstract]) OR "cerebrovascular apoplexy"[Title/Abstract] OR "apoplexy cerebrovascular"[Title/Abstract] OR "vascular accident brain"[Title/Abstract] OR "brain vascular accident"[Title/Abstract] OR "brain vascular accidents"[Title/Abstract] OR (("blood vessels"[MeSH Terms] OR ("blood"[All Fields] AND "vessels"[All Fields]) OR "blood vessels"[All Fields] OR "Vascular"[All Fields] OR "neovascularization, pathologic"[MeSH Terms] OR ("neovascularization"[All Fields] AND "pathologic"[All Fields]) OR "pathologic neovascularization"[All Fields] OR "vascularisation"[All Fields] OR "vascularization"[All Fields] OR "vascularisations"[All Fields] OR "vascularise"[All Fields] OR "vascularised"[All Fields] OR "vascularities"[All Fields] OR "vascularitis"[All Fields] OR "vascularity"[All Fields] OR "vascularizations"[All Fields] OR "vascularize"[All Fields] OR "vascularized"[All Fields] OR "vascularizes"[All Fields] OR "vascularizing"[All Fields] OR "vasculars"[All Fields]) AND "accidents brain"[Title/Abstract]) OR "cerebrovascular stroke"[Title/Abstract] OR "cerebrovascular strokes"[Title/Abstract] OR "stroke cerebrovascular"[Title/Abstract] OR "strokes cerebrovascular"[Title/Abstract] OR "Apoplexy"[Title/Abstract] OR "cerebral stroke"[Title/Abstract] OR "cerebral strokes"[Title/Abstract] OR "stroke cerebral"[Title/Abstract] OR "strokes cerebral"[Title/Abstract] OR "stroke acute"[Title/Abstract] OR "acute stroke"[Title/Abstract] OR "acute strokes"[Title/Abstract] OR "strokes acute"[Title/Abstract] OR "cerebrovascular accident acute"[Title/Abstract] OR "acute cerebrovascular accident"[Title/Abstract] OR "acute cerebrovascular accidents"[Title/Abstract] OR "cerebrovascular accidents acute"[Title/Abstract]

# ("Ultrasonography"[Title/Abstract] OR "diagnostic ultrasound"[Title/Abstract] OR "diagnostic ultrasounds"[Title/Abstract] OR "ultrasound diagnostic"[Title/Abstract] OR "ultrasounds diagnostic"[Title/Abstract] OR "ultrasound imaging"[Title/Abstract] OR "imaging ultrasound"[Title/Abstract] OR (("image"[All Fields] OR "image s"[All Fields] OR "imaged"[All Fields] OR "imager"[All Fields] OR "imager s"[All Fields] OR "imagers"[All Fields] OR "images"[All Fields] OR "Imaging"[All Fields] OR "imaging s"[All Fields] OR "Imagings"[All Fields]) AND "Ultrasound"[Title/Abstract]) OR "Echotomography"[Title/Abstract] OR "ultrasonic imaging"[Title/Abstract] OR "imaging ultrasonic"[Title/Abstract] OR "sonography medical"[Title/Abstract] OR "medical sonography"[Title/Abstract] OR "ultrasonographic imaging"[Title/Abstract] OR "imaging ultrasonographic"[Title/Abstract] OR (("image"[All Fields] OR "image s"[All Fields] OR "imaged"[All Fields] OR "imager"[All Fields] OR "imager s"[All Fields] OR "imagers"[All Fields] OR "images"[All Fields] OR "Imaging"[All Fields] OR "imaging s"[All Fields] OR "Imagings"[All Fields]) AND "Ultrasonographic"[Title/Abstract]) OR "ultrasonographic imagings"[Title/Abstract] OR "Echography"[Title/Abstract] OR "diagnosis ultrasonic"[Title/Abstract] OR "diagnoses ultrasonic"[Title/Abstract] OR "ultrasonic diagnoses"[Title/Abstract] OR "ultrasonic diagnosis"[Title/Abstract] OR "echotomography computer"[Title/Abstract] OR "computer echotomography"[Title/Abstract] OR "tomography ultrasonic"[Title/Abstract] OR "ultrasonic tomography"[Title/Abstract]) AND ("Diaphragm"[Title/Abstract] OR "Diaphragms"[Title/Abstract] OR "respiratory diaphragm"[Title/Abstract] OR "diaphragm respiratory"[Title/Abstract] OR (("Diaphragm"[MeSH Terms] OR "Diaphragm"[All Fields] OR "contraceptive devices, female"[MeSH Terms] OR ("contraceptive"[All Fields] AND "devices"[All Fields] AND "female"[All Fields]) OR "female contraceptive devices"[All Fields] OR "Diaphragms"[All Fields] OR "diaphragm s"[All Fields] OR "diaphragmal"[All Fields]) AND "Respiratory"[Title/Abstract]) OR (("eur med j respir"[Journal] OR "Respiratory"[All Fields]) AND "Diaphragms"[Title/Abstract]) OR ("respiratory muscles"[Title/Abstract] OR "muscle respiratory"[Title/Abstract] OR "muscles respiratory"[Title/Abstract] OR "respiratory muscle"[Title/Abstract] OR "ventilatory muscles"[Title/Abstract] OR "muscle ventilatory"[Title/Abstract] OR "muscles ventilatory"[Title/Abstract] OR "ventilatory muscle"[Title/Abstract]) OR ("intercostal muscles"[Title/Abstract] OR "intercostal muscle"[Title/Abstract] OR "muscle intercostal"[Title/Abstract] OR "muscles intercostal"[Title/Abstract]) OR ("trapezius"[Title/Abstract] OR "sternocleidomastoid muscles"[Title/Abstract] OR "scalene muscles"[Title/Abstract] OR "parasternal intercostal muscle"[Title/Abstract] OR "inspiratory muscle"[Title/Abstract] OR "expiratory muscle"[Title/Abstract]))

("Stroke"[Title/Abstract] OR "Strokes"[Title/Abstract] OR "cerebrovascular accident"[Title/Abstract] OR "cerebrovascular accidents"[Title/Abstract] OR (("Stroke"[MeSH Terms] OR "Stroke"[All Fields] OR "cva"[All Fields]) AND "cerebrovascular accident"[Title/Abstract]) OR ("CVAs"[All Fields] AND "cerebrovascular accident"[Title/Abstract]) OR "cerebrovascular apoplexy"[Title/Abstract] OR "apoplexy cerebrovascular"[Title/Abstract] OR "vascular accident brain"[Title/Abstract] OR "brain vascular accident"[Title/Abstract] OR "brain vascular accidents"[Title/Abstract] OR (("blood vessels"[MeSH Terms] OR ("blood"[All Fields] AND "vessels"[All Fields]) OR "blood vessels"[All Fields] OR "Vascular"[All Fields] OR "neovascularization, pathologic"[MeSH Terms] OR ("neovascularization"[All Fields] AND "pathologic"[All Fields]) OR "pathologic neovascularization"[All Fields] OR "vascularisation"[All Fields] OR "vascularization"[All Fields] OR "vascularisations"[All Fields] OR "vascularise"[All Fields] OR "vascularised"[All Fields] OR "vascularities"[All Fields] OR "vascularitis"[All Fields] OR "vascularity"[All Fields] OR "vascularizations"[All Fields] OR "vascularize"[All Fields] OR "vascularized"[All Fields] OR "vascularizes"[All Fields] OR "vascularizing"[All Fields] OR "vasculars"[All Fields]) AND "accidents brain"[Title/Abstract]) OR "cerebrovascular stroke"[Title/Abstract] OR "cerebrovascular strokes"[Title/Abstract] OR "stroke cerebrovascular"[Title/Abstract] OR "strokes cerebrovascular"[Title/Abstract] OR "Apoplexy"[Title/Abstract] OR "cerebral stroke"[Title/Abstract] OR "cerebral strokes"[Title/Abstract] OR "stroke cerebral"[Title/Abstract] OR "strokes cerebral"[Title/Abstract] OR "stroke acute"[Title/Abstract] OR "acute stroke"[Title/Abstract] OR "acute strokes"[Title/Abstract] OR "strokes acute"[Title/Abstract] OR "cerebrovascular accident acute"[Title/Abstract] OR "acute cerebrovascular accident"[Title/Abstract] OR "acute cerebrovascular accidents"[Title/Abstract] OR "cerebrovascular accidents acute"[Title/Abstract]) AND (("Ultrasonography"[Title/Abstract] OR "diagnostic ultrasound"[Title/Abstract] OR "diagnostic ultrasounds"[Title/Abstract] OR "ultrasound diagnostic"[Title/Abstract] OR "ultrasounds diagnostic"[Title/Abstract] OR "ultrasound imaging"[Title/Abstract] OR "imaging ultrasound"[Title/Abstract] OR (("image"[All Fields] OR "image s"[All Fields] OR "imaged"[All Fields] OR "imager"[All Fields] OR "imager s"[All Fields] OR "imagers"[All Fields] OR "images"[All Fields] OR "Imaging"[All Fields] OR "imaging s"[All Fields] OR "Imagings"[All Fields]) AND "Ultrasound"[Title/Abstract]) OR "Echotomography"[Title/Abstract] OR "ultrasonic imaging"[Title/Abstract] OR "imaging ultrasonic"[Title/Abstract] OR "sonography medical"[Title/Abstract] OR "medical sonography"[Title/Abstract] OR "ultrasonographic imaging"[Title/Abstract] OR "imaging ultrasonographic"[Title/Abstract] OR (("image"[All Fields] OR "image s"[All Fields] OR "imaged"[All Fields] OR "imager"[All Fields] OR "imager s"[All Fields] OR "imagers"[All Fields] OR "images"[All Fields] OR "Imaging"[All Fields] OR "imaging s"[All Fields] OR "Imagings"[All Fields]) AND "Ultrasonographic"[Title/Abstract]) OR "ultrasonographic imagings"[Title/Abstract] OR "Echography"[Title/Abstract] OR "diagnosis ultrasonic"[Title/Abstract] OR "diagnoses ultrasonic"[Title/Abstract] OR "ultrasonic diagnoses"[Title/Abstract] OR "ultrasonic diagnosis"[Title/Abstract] OR "echotomography computer"[Title/Abstract] OR "computer echotomography"[Title/Abstract] OR "tomography ultrasonic"[Title/Abstract] OR "ultrasonic tomography"[Title/Abstract]) AND ("Diaphragm"[Title/Abstract] OR "Diaphragms"[Title/Abstract] OR "respiratory diaphragm"[Title/Abstract] OR "diaphragm respiratory"[Title/Abstract] OR (("Diaphragm"[MeSH Terms] OR "Diaphragm"[All Fields] OR "contraceptive devices, female"[MeSH Terms] OR ("contraceptive"[All Fields] AND "devices"[All Fields] AND "female"[All Fields]) OR "female contraceptive devices"[All Fields] OR "Diaphragms"[All Fields] OR "diaphragm s"[All Fields] OR "diaphragmal"[All Fields]) AND "Respiratory"[Title/Abstract]) OR (("eur med j respir"[Journal] OR "Respiratory"[All Fields]) AND "Diaphragms"[Title/Abstract]) OR ("respiratory muscles"[Title/Abstract] OR "muscle respiratory"[Title/Abstract] OR "muscles respiratory"[Title/Abstract] OR "respiratory muscle"[Title/Abstract] OR "ventilatory muscles"[Title/Abstract] OR "muscle ventilatory"[Title/Abstract] OR "muscles ventilatory"[Title/Abstract] OR "ventilatory muscle"[Title/Abstract]) OR ("intercostal muscles"[Title/Abstract] OR "intercostal muscle"[Title/Abstract] OR "muscle intercostal"[Title/Abstract] OR "muscles intercostal"[Title/Abstract]) OR ("trapezius"[Title/Abstract] OR "sternocleidomastoid muscles"[Title/Abstract] OR "scalene muscles"[Title/Abstract] OR "parasternal intercostal muscle"[Title/Abstract] OR "inspiratory muscle"[Title/Abstract] OR "expiratory muscle"[Title/Abstract])))
