# Supplementary material for: Respiratory muscle ultrasonography evaluation and its clinical application in stroke patients: A review
Source: Front Neurosci. 2023 Apr 6;17:1132335. doi: 10.3389/fnins.2023.1132335 (PMC10115993; doi:10.3389/fnins.2023.1132335)
Supplement: Supplementary file 2 [file Data_Sheet_2.docx]

| Diaphragm | Condition | Values(Mean ± SD) | | Cases | Age | Reference |
| --- | --- | --- | --- | --- | --- | --- |
|  |  | Hemiplegic Side | Non-Hemiplegic Side |  |  |  |
| Thickness(cm) | Inspiration(TLC)/end expiration | 0.40±0.13/0.21±0.02 | 0.54±0.17/0.22±0.02 | 45 | 49.51±12.48 | ^1^ |
|  | End deep inspiration/end expiration | 0.31±0.11/0.24±0.07 | 0.33±0.10/0.23±0.06 | 45 | 61.02 ± 13.66 | ^2^ |
|  | End-inspiration/End-expiration | 0.27±0.03/0.15±0.02(R)  0.31±0.04/0.17±0.03(L) | 0.29±0.03/0.18±0.02(R)  0.33±0.03/0.20±0.03(L) | 20(R)  21(L) | 64.6± 12.4(R)  66.0 ± 10.3(L) | ^3^ |
|  | Maximum inspiration/end- expiration | 0.38±0.08/0.21±0.05(R)  0.37±0.16/0.20±0.06(L) | 0.48±0.19/0.23±0.05(R)  0.47±0.14/0.21±0.02(L) | 12(R)  13(L) | 47.58±13.00(R)  52.53±9.06(L) | ^4^ |
|  | Calm end-expiratory/maximum end-inspiratory | 0.20±0.12/0.27±0.20  0.19±0.13/0.26±0.19 | 0.23±0.17/0.34±0.31  0.23±0.17/0.35±0.30 | 30  30 | 59.13±11.55  62.27±13.17 | ^5^ |
| Thickening fraction(%) | Inspiration(TLC)/end expiration | 19±12 | 32±17 | 45 | 49.51±12.48 | ^1^ |
|  | End-inspiration/end-expiration | 32.95 ± 36.85 | 43.42 ± 27.62 | 45 | 61.02 ± 13.66 | ^2^ |
|  | Maximum end-inspiratory/end-expiratory | 34.19±6.32  34.29±3.89 | 48.99±6.39  50.10±7.12 | 30  30 | 59.13±11.55  62.27±13.17 | ^5^ |
| Excursion(cm) | Quiet /deep breathing | 1.31±0.54/3.17±1.52 | 1.44±0.50/4.02±1.47 | 45 | 61.02 ± 13.66 | ^2^ |
|  | Spontaneous/deep respiration  Quiet breathing/deep breathing | 1.8±0.7/5.1±2.2(R)  1.8±0.7/5.1±2.2(L) | 2.2±0.7/4.9±1.9(R)  2.2±0.7/4.9±1.9(L) | 15(R)  8(L) | 58.8±11.6(R)  63.6±8.3(L) | ^6^  ^7^ |
|  | Quiet /deep breathing | 1.27±0.11/2.75±0.40  1.25 0.10/ 2.77 0.41 | 1.46±0.13/ 5.08 0.68  1.41 0.13/ 4.96 0.62 | 30  30 | 59.13±11.55  62.27±13.17 | ^5^ |

| Thickness | Condition | Values (Mean ± SD) | | Cases | Age | Reference |
| --- | --- | --- | --- | --- | --- | --- |
|  |  | Hemiplegic Side | Non-Hemiplegic Side |  |  |  |
| IO (cm) |  |  |  |  |  |  |
|  | The end of relaxed expiration | 0.67±0.23 | 0.64±0.20 | 32 | 71.3±10.2 | ^8^ |
|  | The resting and contraction states | 0.648±0.209 | 0.67±0.221 | 19(H)/36(L) | 70.00±15.28 (H)/61.97±12.32 (L) | ^9^ |
|  | At the start of expiration | 0.3±0.1 |  | 11 | 69.7±6.8/71.6±7.9 | ^10^ |
|  | End of expiration | 0.50 ± 0.13 | 0.52 ± 0.13 | 33 | 58.94 ± 12.30 | ^11^ |
| EO (cm) |  |  |  |  |  |  |
|  | At the end of relaxed expiration | 0.40±0.16 | 0.45±0.15 | 32 | 71.3±10.2 | ^8^ |
|  | The resting and contraction states | 0.356±0.104 | 0.36±0.122 | 19(H)/36(L) | 70.00±15.28 (H)/61.97±12.32 (L) | ^9^ |
|  | End of expiration | 0.23 ± 0.07 | 0.26 ± 0.07 | 33 | 58.94 ± 12.30 | ^11^ |
| TrA (cm) |  |  |  |  |  |  |
|  | The end of relaxed expiration | 0.27±0.09 | 0.29±0.09 | 32 | 71.3±10.2 | ^8^ |
|  | The resting and contraction states | 0.3±0.129 | 0.316±0.135 | 19(H)/36(L) | 70.00±15.28 (H)/61.97±12.32 (L) | ^9^ |
|  | At rest(no clear) | 0.318±0.092 | 0.282±0.099 | 9 | 71.87 ± 8.42 | ^12^ |
|  | At the start of expiration | 0.4±0.2 |  | 11 | 69.7±6.8/71.6±7.9 | ^10^ |
|  | End of expiration | 0.18 ± 0.05 | 0.21 ± 0.05 | 33 | 58.94 ± 12.30 | ^11^ |
| RA (cm) |  |  |  |  |  |  |
|  | The end of relaxed expiration | 0.73±0.21 | 0.75±0.20 | 32 | 71.3±10.2 | ^8^ |
|  | The resting and contraction states | 0.749±0.242 | 0.76±0.226 | 19(H)/36(L) | 70.00±15.28 (H)/61.97±12.32 (L) | ^9^ |
|  | End of expiration | 0.57 ± 0.20 | 0.55 ± 0.20 | 33 | 58.94 ± 12.30 | ^11^ |

**References:**

1. Kim M, Lee K, Cho J, Lee W. Diaphragm thickness and inspiratory muscle functions in chronic stroke patients. *Med Sci Monitor*. 2017;23:1247-1253

2. Liu X, Qu Q, Deng P, Zhao Y, Liu C, Fu C, Jia J. Assessment of diaphragm in hemiplegic patients after stroke with ultrasound and its correlation of extremity motor and balance function. *Brain Sciences*. 2022;12:882

3. Kılıçoğlu MS, Yurdakul OV, Çelik Y, Aydın T. Investigating the correlation between pulmonary function tests and ultrasonographic diaphragm measurements and the effects of respiratory exercises on these parameters in hemiplegic patients. *Top Stroke Rehabil*. 2022;29:218-229

4. Cho JE, Lee HJ, Kim MK, Lee WH. The improvement in respiratory function by inspiratory muscle training is due to structural muscle changes in patients with stroke: A randomized controlled pilot trial. *Top Stroke Rehabil*. 2018;25:37-43

5. Cao H, Chen X, Ren X, Chen Z, Liu C, Ni J, Liu H, Fan Y, Xu D, Jin H, Bao J, Yulun H, Su M. Repetitive transcranial magnetic stimulation combined with respiratory muscle training for pulmonary rehabilitation after ischemic stroke—a randomized, case-control study. *Front Aging Neurosci*. 2022;14

6. VOYVODA N, YÜCEL C, KARATAS G, OGUZULGEN I, OKTAR S. An evaluation of diaphragmatic movements in hemiplegic patients. *Brit J Radiol*. 2012;85:411-414

7. Jung K, Park J, Hwang D, Kim J, Kim J. Ultrasonographic diaphragmatic motion analysis and its correlation with pulmonary function in hemiplegic stroke patients. *Annals of Rehabilitation Medicine*. 2014;38:29

8. Monjo H, Fukumoto Y, Asai T, Shuntoh H. Muscle thickness and echo intensity of the abdominal and lower extremity muscles in stroke survivors. *J Clin Neurol*. 2018;14:549

9. Kim Y, Kim J, Nam H, Kim HD, Eom MJ, Jung SH, Han N. Ultrasound imaging of the trunk muscles in acute stroke patients and relations with balance scales. *Annals of Rehabilitation Medicine*. 2020;44:273-283

10. Oh D, Kim G, Lee W, Shin MM. Effects of inspiratory muscle training on balance ability and abdominal muscle thickness in chronic stroke patients. *J Phys Ther Sci*. 2016;28:107-111

11. Lee K, Cho J, Hwang D, Lee W. Decreased respiratory muscle function is associated with impaired trunk balance among chronic stroke patients: A cross-sectional study. *The Tohoku Journal of Experimental Medicine*. 2018;245:79-88

12. Kelli A, Kellis E, Galanis N, Dafkou K, Sahinis C, Ellinoudis A. Transversus abdominis thickness at rest and exercise in individuals with poststroke hemiparesis. *Sports*. 2020;8:86
